# Analysis of Plasma Metabolic Profile on Ganglion Cell–Inner Plexiform Layer Thickness With Mortality and Common Diseases

**DOI:** 10.1001/jamanetworkopen.2023.13220

**Published:** 2023-05-16

**Authors:** Shaopeng Yang, Zhuoting Zhu, Yixiong Yuan, Shida Chen, Xianwen Shang, Gabriella Bulloch, Mingguang He, Wei Wang

**Affiliations:** 1 State Key Laboratory of Ophthalmology, Zhongshan Ophthalmic Center, Sun Yat-sen University, Guangdong Provincial Key Laboratory of Ophthalmology and Visual Science, Guangdong Provincial Clinical Research Center for Ocular Diseases, Guangzhou, China; 2 Centre for Eye Research Australia, Royal Victorian Eye and Ear Hospital, Melbourne, Victoria, Australia; 3 Department of Ophthalmology, Guangdong Academy of Medical Sciences, Guangdong Provincial People’s Hospital, Guangzhou, China

## Abstract

**Importance:**

The neural retina is considered a unique window to systemic health, but its biological link with systemic health remains unknown.

**Objective:**

To investigate the independent associations of retinal ganglion cell–inner plexiform layer thickness (GCIPLT) metabolic profiles with rates of mortality and morbidity of common diseases.

**Design, Setting, and Participants:**

This cohort study evaluated UK Biobank participants enrolled between 2006 and 2010, and prospectively followed them up for multidisease diagnosis and mortality. Additional participants from the Guangzhou Diabetes Eye Study (GDES) underwent optical coherence tomography scanning and metabolomic profiling and were included for validation.

**Main Outcomes and Measures:**

Systematic analysis of circulating plasma metabolites to identify GCIPLT metabolic profiles; prospective associations of these profiles with mortality and morbidity of 6 common diseases with their incremental discriminative value and clinical utility.

**Results:**

Among 93 838 community-based participants (51 182 [54.5%] women), the mean (SD) age was 56.7 (8.1) years and mean (SD) follow-up was 12.3 (0.8) years. Of 249 metabolic metrics, 37 were independently associated with GCIPLT, including 8 positive and 29 negative associations, and most were associated with the rates of future mortality and common diseases. These metabolic profiles significantly improved the models for discriminating type 2 diabetes over clinical indicators (C statistic: 0.862; 95% CI, 0.852-0.872 vs clinical indicators only, 0.803; 95% CI, 0.792-0.814; *P* < .001), myocardial infarction (0.792; 95% CI, 0.775-0.808 vs 0.768; 95% CI, 0.751-0.786; *P* < .001), heart failure (0.803; 95% CI, 0.786-0.820 vs 0.790; 95% CI, 0.773-0.807; *P* < .001), stroke (0.739; 95% CI, 0.714-0.764 vs 0.719; 95% CI, 0.693-0.745; *P* < .001), all-cause mortality (0.747; 95% CI, 0.734-0.760 vs 0.724; 95% CI, 0.711-0.738; *P* < .001), and cardiovascular disease mortality (0.790; 95% CI, 0.767-0.812 vs 0.763; 95% CI, 0.739-0.788; *P* < .001). Additionally, the potential of GCIPLT metabolic profiles for risk stratification of cardiovascular diseases were further confirmed in the GDES cohort using a different metabolomic approach.

**Conclusions and Relevance:**

In this prospective study of multinational participants, GCIPLT-associated metabolites demonstrated the potential to inform mortality and morbidity risks. Incorporating information on these profiles may facilitate individualized risk stratification for these health outcomes.

## Key Points

**Question:**

Are there associations between retina-related biological changes and mortality and morbidity of common diseases?

**Findings:**

In this cohort study of health information from 93 838 adults in the United Kingdom verified with data from 592 Chinese patients, retinal ganglion cell–inner plexiform layer thickness metabolic profile was associated with mortality and morbidity of 6 common diseases. These profiles improved estimates that incorporated clinical indicators only.

**Meaning:**

These results suggest that retinal ganglion cell–inner plexiform layer thickness metabolic profiles may inform mortality and common disease risks, providing a distinctive insight into the retina as a window to systemic health.

## Teaser

This cohort study of health data from British adults that was validated with a cohort of Chinese adults examines the use of using retinal ganglion cell–inner plexiform layer thickness metabolic profiles to estimate risk of mortality and 6 common diseases vs clinical indicators.

## Introduction

The neural retina is an extension of the central nervous system and a unique window to systemic health.^1,2,3^ The state-of-the-art in vivo optical coherence tomography (OCT) has identified retinal nerve fiber layers (RNFL) and ganglion cell–inner plexiform layer (GCIPL) as biomarkers of aging and various common diseases.^4,5,6,7,8,9,10,11,12,13^ With the booming OCT deployment in primary care settings, risk-free retinal scans have recently become an attractive alternative for screening systemic health in routine community scenarios.^14,15,16^

However, the biological link between neuroretinal alterations and systemic health remains unknown. Metabolomics offers a novel opportunity for the biological profiles underlying these complex features, especially considering that metabolic factors contribute substantially to various common diseases.^17,18,19,20^ Previous studies have reported associations between circulating metabolites and alterations in neuroretinas, but these are limited to single-biomarker approaches.^21,22,23^ Additionally, these studies majorly focused on the RNFL (representing axons of retinal ganglion cells [RGCs]), while growing evidence suggesting that GCIPL (representing cytosol and dendrites of RGCs) is a more sensitive and reproducible mirror for neuroretinal damage and systemic health.^6,24,25,26^

We hypothesized that circulating metabolites may underlie the links between neuroretinal changes and systemic health. The objectives of this study were (1) to identify the metabolic profiles on GCIPL thickness (GCIPLT) in the European population; (2) to assess the associations of GCIPLT-related profiles with risks of mortality and systematic diseases; (3) to evaluate the robustness by employing an independent Chinese cohort with different metabolome-profiling approach.

## Methods

### Study Design and Participants

The UK Biobank (UKB) study is a large population-based multicenter prospective cohort study including over half a million participants aged 40 to 69 years from England, Scotland, and Wales registered with the National Health Service (NHS) in 2006 to 2010.^27^ The Guangzhou Diabetes Eye Study (GDES) is a community-based cohort study that recruits 2300 patients with type 2 diabetes aged 35 to 85 years from 2017 through 2019 in Guangzhou, China.^28^ Data collection was conducted from March 2006 to March 2021 for the UKB cohort and November 2017 to December 2022 for the GDES cohort. This study was conducted in accordance with the principles of the Declaration of Helsinki,^29^ and was approved by the North West Multi-Center Research Ethics Committee and the Ethics Committee of Zhongshan Ophthalmic Center. All participants signed an informed consent form. This study follows the Strengthening the Reporting of Observational Studies in Epidemiology (STROBE) reporting guidelines.

The current study consisted of multiple consecutive phases (eFigure 1 and eFigure 2 in Supplement 1). In phase 1, we performed a systematic analysis of circulating plasma metabolomics data from 7824 participants in the UKB cohort to identify GCIPLT metabolic profiles. In phase 2, a prospective cohort study was conducted on a nonoverlapping subset of 86 014 participants who were followed up for incident end point events to assess the association of these profiles with each of the 10 study end points, as well as their added discriminatory and clinical utility (eMethods in Supplement 1). The end point events were incident type 2 diabetes, myocardial infarction, heart failure, stroke, dementia, obstructive sleep apnea or hypopnea syndrome (OSAHS), and mortality. Mortality was further classified into all-cause, cardiovascular disease (CVD), cancer, and other mortality. Finally, we tested whether GCIPLT metabolic profiles informed CVD risk in an independent population from southern China using a different metabolomic approach.

### Proton Nuclear Magnetic Resonance Metabolomics in UKB

Metabolomics profiling was conducted using a high-throughput proton nuclear magnetic resonance (1H-NMR) platform (Nightingale Health). Detailed protocols are described elsewhere,^30^ but in brief, cryopreserved plasma samples were thawed and centrifuged, and the supernatant was mixed with phosphate buffer. The samples were then loaded onto a cooled sample changer, and 2 NMR spectra of each plasma sample were recorded using a 500 MHz NMR spectrometer (Bruker). After stringent quality control (eMethods in Supplement 1), the metabolic metrics were quantified using the Nightingale Health biomarker quantification library 2020, including 168 metrics presented at absolute levels (ie, fatty acids, glycolytic metabolites, ketone bodies, amino acids, lipids, and lipoproteins) and 81 metrics presented as ratio values (eTable 1 in Supplement 1).

### Optical Coherence Tomography Imaging in UKB

Retina spectral-domain OCT was performed in a closed darkroom using an OCT scanner (Topcon, Inc). The system had an axial resolution of 6 μm and an image acquisition rate of 18 000 A-scans per second. Using a 3-dimensional 6 × 6 mm macular volume scan mode, the retina was imaged at a scan density of 512 A-scans by 128 B-scans in 3.6 seconds. The Topcon Advanced Boundary Segmentation algorithm version 1.6.1.1 (Topcon, Inc) automatically segmented the retinal layers and determined the GCIPLT. Image quality score, internal limiting membrane indicator, validity count, and motion indicators were used to detect and ensure quality control, whereby images with low signal strength (Q below 45) or poor segmentation or centration (poorest 20% of each indicator) were excluded (eMethods in Supplement 1). If both eyes were eligible for the analysis, 1 eye was randomly selected for further analysis.

### Assessments of Outcomes and Covariates in UKB

The Hospital Episode Statistics database, Scottish Morbidity Record, and Patient Episode Database were used to record inpatient hospital records for England, Scotland, and Wales (eMethods in Supplement 1). Mortality data were obtained from national data sets with the NHS Digital (England and Wales) and NHS Central Register (Scotland). The determination of common diseases and the recording of primary cause of mortality were based on the *International Statistical Classification of Diseases and Related Health Problems, Tenth Revision (ICD-10)*. The follow-up period was from March 16, 2006, to March 31, 2021. Person-days for each participant were calculated from the date of baseline assessment to the date of disease onset, mortality, or the end of follow-up, whichever came first. Physical measurements, face-to-face interviews, and detailed self-administered touchscreen questionnaires were conducted on all participants at baseline.

### Validation in the GDES Cohort

A total of 592 participants from the GDES cohort who met similar eligibility criteria as participants from the UKB were included for analysis. At baseline, all participants underwent retinal swept-source OCT (Topcon) scanning, which employed a 3D Macula Cube 7 × 7 mm–volume scan mode centered at the fovea for GCIPLT measurements (eMethods in Supplement 1). Additionally, they also underwent liquid chromatography tandem triple quadrupole mass spectrometry (SCIEX) for metabolomics profiling. Incident CVD was defined as the development of coronary heart disease, heart failure, atrial fibrillation, stroke, or related mortality during the follow-up period, determined by a combination of medical records, standard questionnaires, and verbal interviews.

### Statistical Analysis

R software version 4.2.2 (R Project for Statistical Computing) was used for all data analyses and presentation of results. Continuous variables were compared using the *t* test or the Mann-Whitney U test, as appropriate. Categorical variables were compared using χ^2^ test. *Z*-score normalization was applied to all metabolic metrics for comparability.

In phase 1, the associations between metabolites and GCIPLT were assessed using multilevel linear regression models after adjusting for age, sex, race, education, Townsend deprivation index, household income, body mass index (BMI; calculated as weight in kilograms divided by height in meters squared), smoking status, alcohol consumption status, use of lipid-lowering medications, spherical equivalent, and intraocular pressure (eFigure 3 in Supplement 1). The Benjamini-Hochberg method was employed to reduce the false-positive rate. In phase 2, participants were randomly divided at a ratio of 7:3 into discovery and validation sets. Participants with previous diagnosis were excluded for each analysis (eg, in the case of type 2 diabetes end point analysis, participants diagnosed with type 2 diabetes at baseline were excluded). The GCIPLT-associated metabolites were analyzed using Cox proportional hazard models for the rates of 6 common diseases and 4 mortality types, adjusting for age, sex, race, education, Townsend deprivation coefficient, household income, BMI, smoking status, alcohol consumption status, and use of lipid-lowering medications. The proportional hazard assumption and linearity assumption were tested using the Schoenfeld residual method and the Martingale residuals method, respectively. The interaction terms with time were implemented in the models when necessary (eMethods in Supplement 1).

We developed 3 models to discriminate each outcome in the discovery set, namely a clinical indicators–based model, a GCIPLT metabolic state model, and a combined model, and the performance of each was then evaluated in the validation set (eMethods, eTable 2 in Supplement 1). The event rate ratios between individuals in the top, middle, and bottom deciles of the GCIPLT metabolic states were calculated to enable comparison among different health outcomes.^31^ To assess the added discrimination of these profiles in comparison with other clinical indicators, we calculated Harrell C statistics. Net reclassification indices were then calculated to quantify the net benefits in the reclassification ability of adding these profiles to the clinical indicators-based models. Calibration plots were built to assess the goodness of model fit. Finally, we performed decision curve analyses to estimate the benefits in clinical utility (eMethods in Supplement 1). A *P* value <.05 was statistically significant in 2-sided tests, with exceptions where specified.

## Results

### Baseline Characteristics

Among 93 838 participants (51 182 [54.5%] women), the mean (SD) age was 56.7 (8.1) years. A total of 7824 eyes of 7824 participants (population 1) were eligible for phase 1 analysis. For the phase 2 analysis, 86 014 participants were eligible (population 2). Participants who underwent OCT scanning at baseline were younger, male, more educated, had a higher income, had a lower BMI, smoked less, and were less likely to be on lipid-lowering or antihypertensive medications than those who did not. The distributions of participant characteristics in the discovery and validation sets were similar (all *P* > .05) (eTable 3 and 4 in Supplement 1).

### Metabolites Associated With GCIPLT

Of the 249 metabolic metrics, 37 reached significances for multiple comparisons, including 29 with negative associations, covering phospholipids, total lipids, cholesterol, and cholesteryl esters in high-density lipoprotein (HDL), apolipoprotein A1 (apoA1), cholines, glucose, and saturated fatty acids, with adjusted β values ranging from −0.403 per 1-SD change (95% CI, −0.568 to −0.238) for the ratio of saturated fatty acids to total fatty acids to −0.160 per 1-SD change (95% CI, −0.287 to −0.032) for the triglycerides to total lipids ratio in small very low–density lipoprotein. In contrast, the ratios of linoleic acid to total fatty acids (adjusted β value per 1-SD change, 0.324; 95% CI, 0.174-0.475), omega-6 to total fatty acids (0.258; 95% CI, 0.109-0.408), and apolipoprotein B (apoB) to apoA1 (0.221; 95% CI, 0.091-0.350), were positively associated with GCIPLT, in addition to several low- and very low–density lipoprotein components (eTable 5 in Supplement 1).

### Metabolic Profiles and Morbidity and Mortality Risks

In phase 2, 6524 participants (7.3%) died at a median (IQR) follow-up time of 12.3 (11.6-13.0) years. Of these, 1544 (1.8%) died of cardiovascular causes, 3151 (3.7%) of cancer, and 1559 (1.8%) of other causes (eTable 6 in Supplement 1). A total of 6071 participants (6.5%) developed type 2 diabetes, 2866 (3.1%) developed myocardial infarction, 2537 (2.7%) developed heart failure, 1578 (1.7%) developed stroke, 1219 (1.3%) developed dementia, and 1366 (1.5%) developed OSAHS.

The associations of GCIPLT metabolic profiles with each outcome were summarized in eFigure 4 in Supplement 1. Type 2 diabetes, myocardial infarction, OSAHS, and all-cause mortality were each associated independently with over 30 of these profiles. Heart failure, stroke, dementia, CVD mortality, cancer mortality, and other mortality were also associated with at least 20 of these profiles.

**Figure 1.**
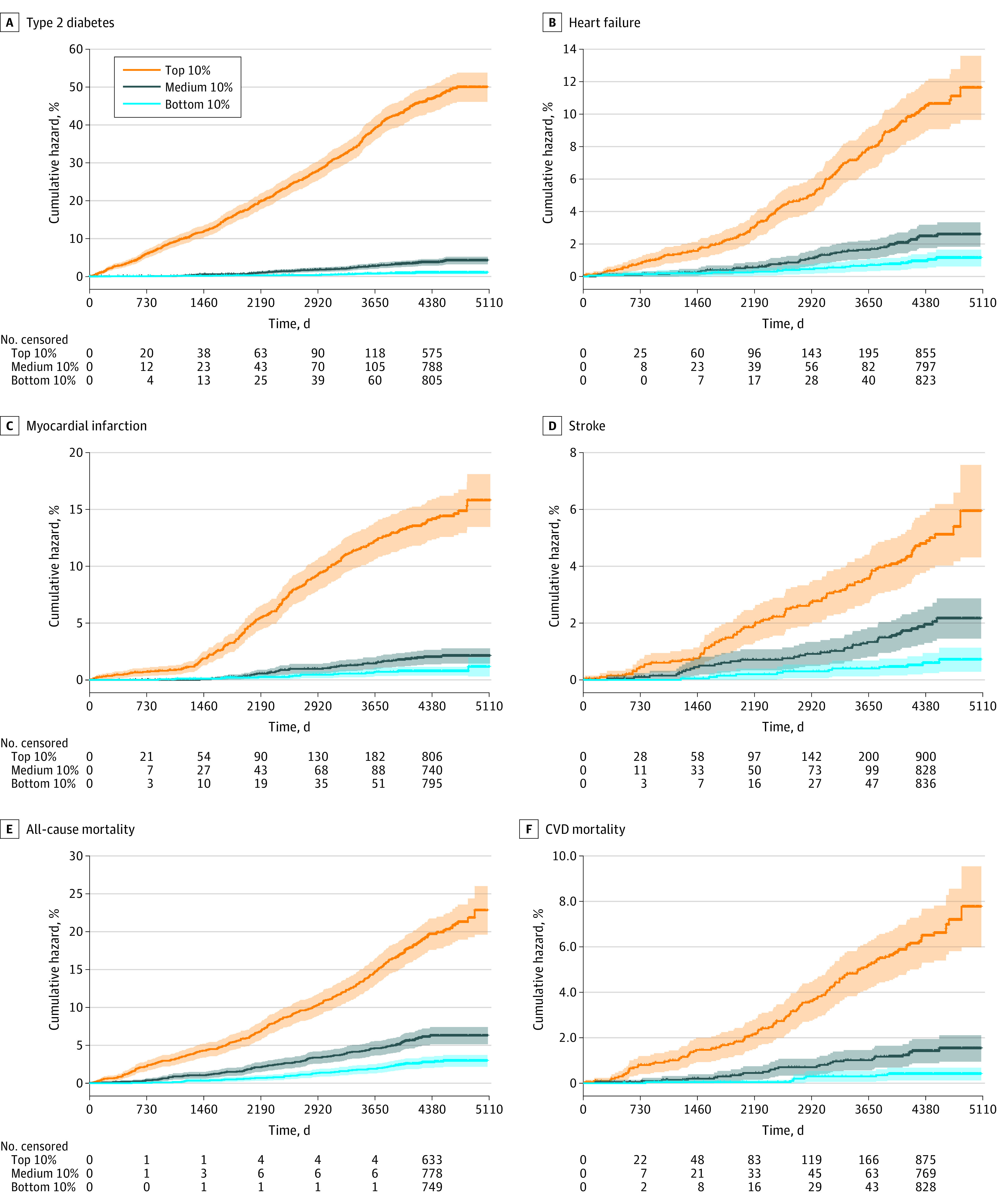
Cumulative Event Rates for Common Diseases and Mortality Stratified by GCIPLT Metabolic State Quantiles An extended figure displaying all study outcomes is available in eFigure 5 in Supplement 1. GCIPLT indicates ganglion cell-inner plexiform layer thickness; CVD, cardiovascular disease.

Increasing event rates over GCIPLT metabolic states were observed for all health outcomes (Figure 1; eFigure 5 in Supplement 1). Participants in the top 10% of GCIPLT metabolic states had event rates more than 10-fold higher compared with those in the bottom 10% for type 2 diabetes (hazard ratio [HR], 44.36; 95% CI, 28.45-69.18), myocardial infarction (HR, 17.78; 95% CI, 10.73-29.47), dementia (HR, 11.15; 95% CI, 5.41-23.00), and CVD mortality (HR, 12.08; 95% CI, 6.33-23.04), suggesting the rich information that GCIPLT metabolic states hold in these outcomes. Ratios were great than 5-fold higher for stroke (HR, 9.15; 95% CI, 4.76-17.59), heart failure (HR, 8.62; 95% CI, 5.54-13.41), OSAHS (HR, 8.82; 95% CI, 4.96-15.66), all-cause (HR, 5.60; 95% CI, 5.32-9.34), and other mortality (HR, 5.60; 95% CI, 3.39-9.25). While modest, GCIPLT metabolic state-stratified risk trajectories also separated cancer mortality risk (HR, 4.50; 95% CI, 3.10-6.53).

### Improvements in Discriminatory and Clinical Utility Over Clinical Indicators

**Figure 2.**
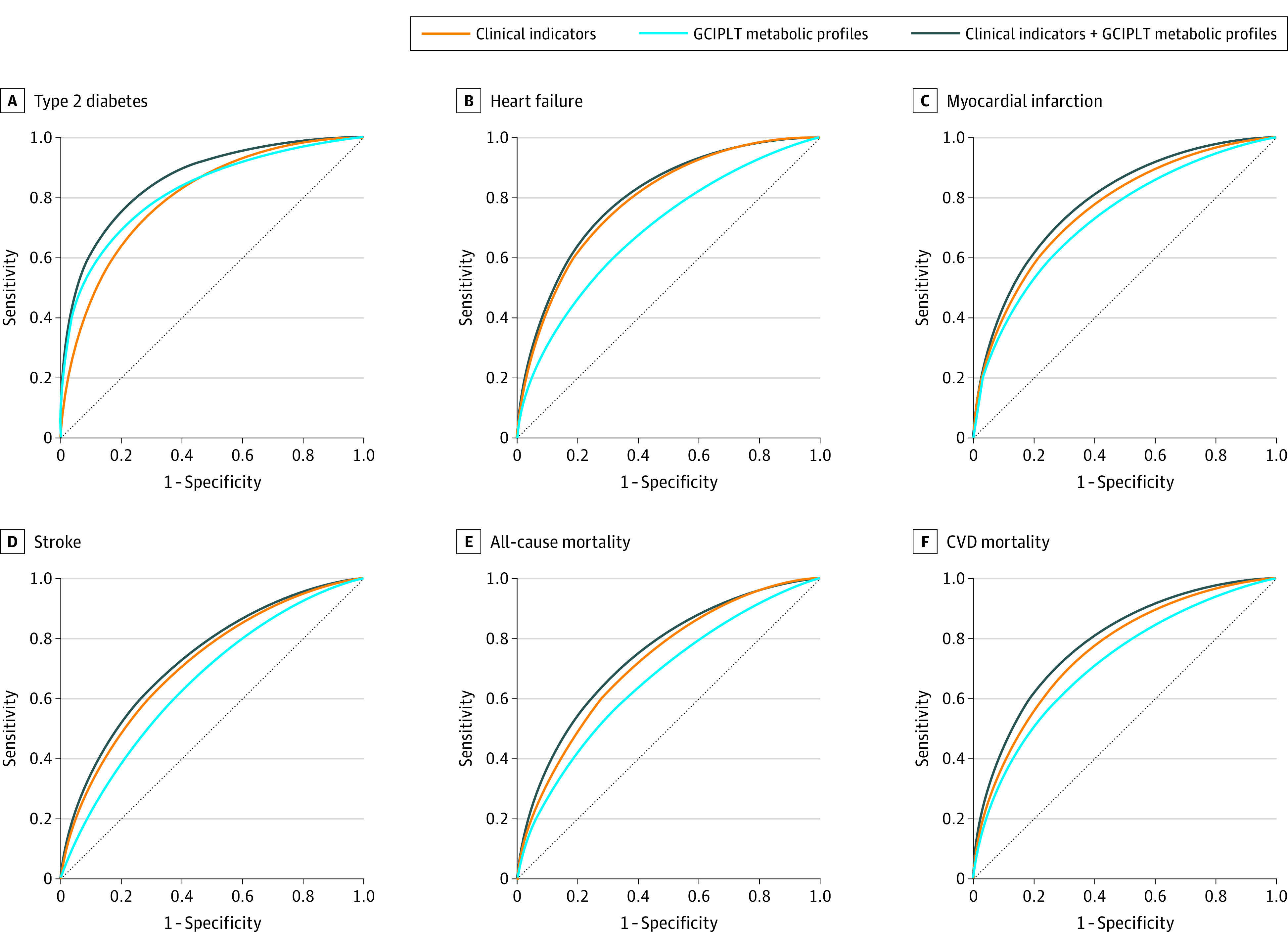
Receiver Operating Characteristic Curves of Clinical Indicator, GCIPLT Metabolic State, and Combined Models for Predicting Common Diseases and Mortality An extended figure displaying all study outcomes is available in eFigure 7 in Supplement 1. GCIPLT indicates ganglion cell-inner plexiform layer thickness; CVD, cardiovascular disease.

**Figure 3.**
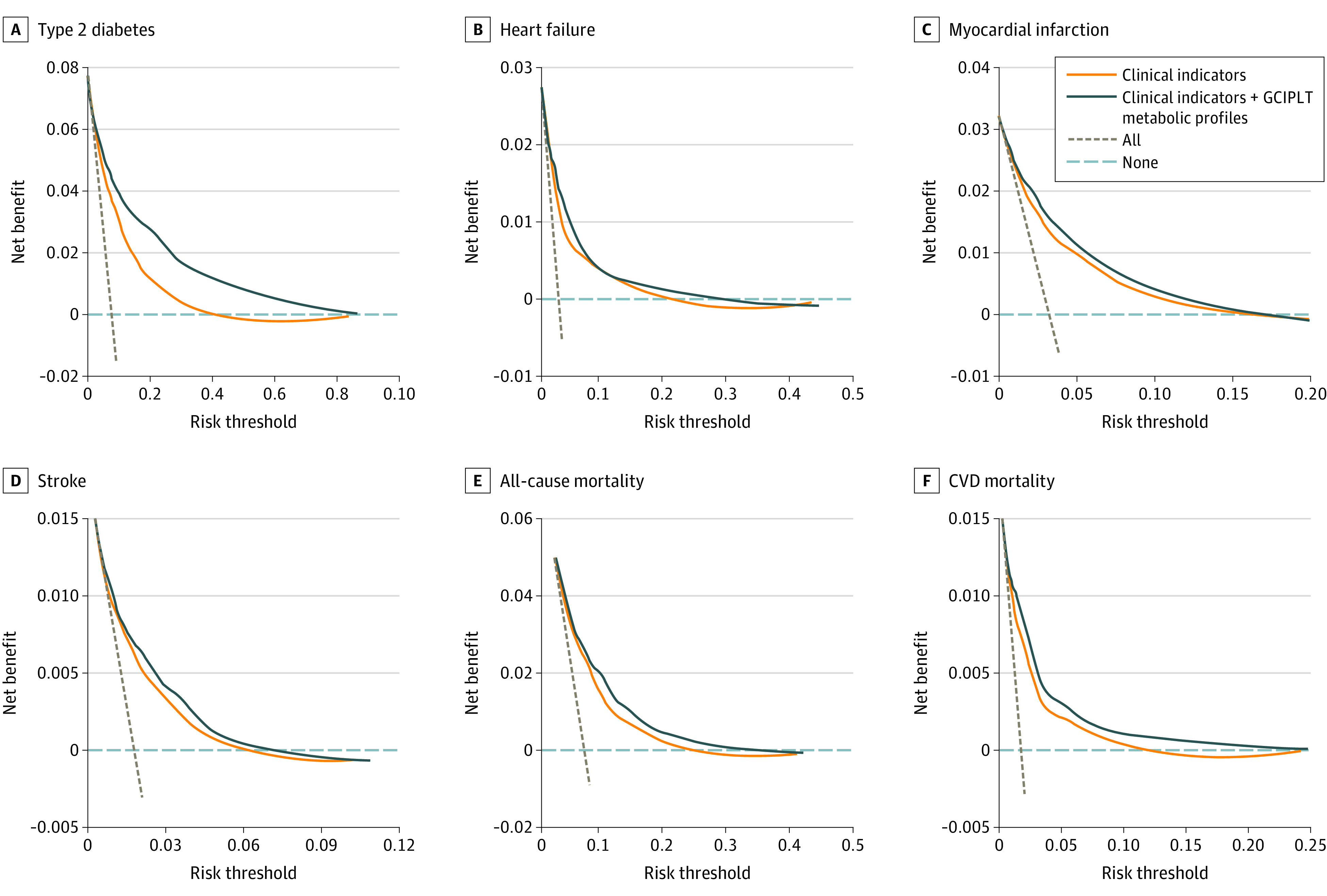
Net Benefit Curves of Clinical Utility for Common Diseases and Mortality An extended figure displaying all study outcomes is available in eFigure 9 in Supplement 1. Horizontal dashed gray lines indicate treat none, and vertical dashed brown lines indicate treat all; GCIPLT, ganglion cell-inner plexiform layer thickness; CVD, cardiovascular disease.

GCIPLT metabolomic profiles showed discriminative value higher than or comparable with that of all other clinical indicators, including age, for type 2 diabetes, heart failure, myocardial infarction, stroke, OSAHS, CVD mortality, and other mortality (eFigure 6 in Supplement 1). Adding these profiles resulted in an increase in C statistics for type 2 diabetes (0.862; 95% CI, 0.852-0.872 vs clinical indicators only, 0.803; 95% CI, 0.792-0.814; *P* < .001), heart failure (0.803; 95% CI, 0.786-0.820 vs 0.790; 95% CI, 0.773-0.807; *P* < .001), myocardial infarction (0.792; 95% CI, 0.775-0.808 vs 0.768; 95% CI, 0.751-0.786; *P* < .001), stroke (0.739; 95% CI, 0.714-0.764 vs 0.719; 95% CI, 0.693-0.745; *P* < .001), OSAHS (0.758; 95% CI, 0.734-0.783 vs 0.748; 95% CI, 0.719; *P* = .02), CVD mortality (0.790; 95% CI, 0.767-0.812 vs 0.763; 95% CI, 0.739-0.788; *P* < .001) (Figure 2; eFigure 7 and eTable 7 in Supplement 1). Significant net reclassification index improvements were also observed (eTable 8 in Supplement 1). Calibration was evaluated for models of all health outcomes (eFigure 8 in Supplement 1), and decision curve analyses confirmed further improvements in the clinical utility with the addition of GCIPLT metabolic profiles (Figure 3; eFigure 9 in Supplement 1).

### Extrapolation in the GDES Cohort

**Figure 4.**
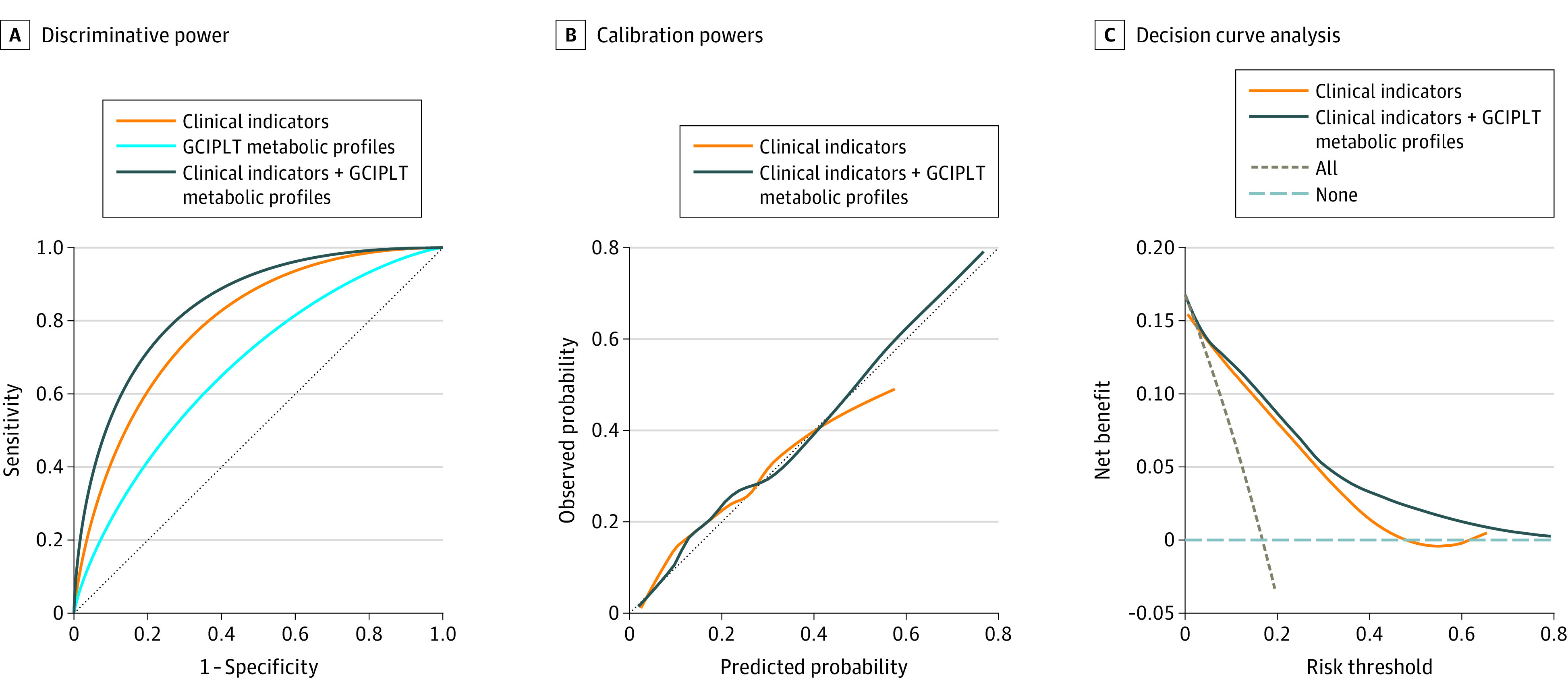
Improvements in Predictability, Calibration, and Clinical Utility of Incorporating Metabolic Profiles for CVD Stratification in the GDES Cohort CVD indicates cardiovascular disease; GCIPLT, ganglion cell-inner plexiform layer thickness; GDES, Guangzhou Diabetes Eye Study.

We then evaluated the potential for GCIPLT metabolic profiles to discriminate CVDs, a group of diseases with strong metabolomic contributions over the comprehensive clinical indicators, in the GDES cohort using liquid chromatography–mass spectrometry assays. Of the 592 participants with type 2 diabetes, 99 (16.7%) experienced a CVD event during the 4-year follow-up. Using liquid chromatography–mass spectrometry assays, 24 plasma metabolites were identified as GCIPLT metabolic profiles (eTable 9 in Supplement 1), and the incorporation of these profiles significantly improved the discriminative performance over clinical indicators for CVD stratification, while the improved calibration and clinical utility were also confirmed (Figure 4).

## Discussion

We identified GCIPLT metabolic profiles in the European general population and, to our knowledge, provided the first evidence for their prospective association with mortality and morbidity of 6 common diseases. These profiles informed mortality and morbidity risks, and helped improve the discriminative performance and clinical utility for common health outcomes over clinical indicators. The potential of GCIPLT metabolic profiles for CVD risk stratification was further confirmed in an independent cohort using a different metabolomic approach. These findings highlight the value of GCIPLT-associated metabolites for individualized risk stratification of mortality and common diseases, providing a distinctive insight into the retina as a window to systemic health.

Among the 37 GCIPLT-associated metabolites identified in phase 1, most were protective against all- and specific-cause mortality, reinforcing their roles in systemic health status and various disease processes in the human body. While age was typically the strongest estimator for mortality, it was noteworthy that the performance of these profiles for discriminating CVD mortality was even greater than age. This may be attributed to the fact that these profiles were found associated with various CVDs in the current study, including myocardial infarction, heart failure, and stroke. Furthermore, the significant improvement in discriminative value and clinical utility for cancer and other mortality suggests the potential for these profiles to characterize much broader systemic health states.

CVDs are the leading cause of death worldwide, and their risk assessment is a critical component of prevention.^32^ Our study revealed that GCIPLT metabolic profiles significantly affected various CVDs and related mortality rates, highlighting the promising role of these profiles in risk stratification and prevention of CVDs. These profiles included apoA1, cholesteryl esters, free cholesterol, phospholipids, and phosphatidylcholines, and were believed to play a critical role in the efflux of cellular cholesterol through HDL, thereby preventing the accumulation of oxidized lipids and reducing vascular inflammation.^33,34^ In addition, linoleic acid and omega-6 fatty acids were also protective against multiple CVDs and related mortality, which is intriguing considering that the role of omega-6 fatty acids in CVDs remains debated.^35,36^ Recent large-scale prospective studies supported the protective value of circulating linoleic acid against CVDs,^37^ and given that it also protected against type 2 diabetes, OSAHS, dementia, and various mortality types in the current study, it is plausible that the substance may provide a broader protective effect on systemic health. An extended discussion of other end points in this study is available in eAppendix in Supplement 1.

Given the low sensitivity of 1H-NMR assays, we further employed liquid chromatography–mass spectrometry metabolomic assays to validate the value of GCIPLT-related metabolites for CVD risk stratification in a southern Chinese population. With this approach, additional GCIPLT metabolic profiles were further identified, spanning a wide range of nucleotides, amino acids, organic acids, and heterocyclic compounds. It is also worth noting that swept-source OCT scanning was performed in the GDES cohort, which offers improved depth resolution and reduced imaging artifacts compared with conventional spectral domain OCT, thereby providing more accurate GCIPLT measurements.^38^ Overall, the significant improvements in CVD risk stratification in the southern Chinese diabetic population further confirmed the potential of these profiles to capture common disease risks in a much broader population. However, both NMR-based and mass spectronomy–based metabolomics profiling provide only a partial snapshot of the plasma metabolic phenotype. Higher-coverage metabolomic approaches or the combination of multiple approaches would be necessary to provide a more comprehensive GCIPLT metabolic landscape in the future to improve the system-level understanding of this ocular biomarker that indicates systemic health.

### Limitations

This study had several limitations. First, some patients lacked diagnostic data in their initial hospital records; therefore, their diagnosis were based on self-reported questionnaires, which may have introduced misclassification. Second, there were certain differences in the baseline characteristics of participants who underwent retinal OCT measurements compared with others. Therefore, caution should be exercised before generalizing the metabolic profiles to a more general population. Third, the metabolomics profiling was based on a single sample collection at baseline and therefore may not reflect the fluctuations of these metabolites over time. Fourth, it is important to note that validation of the exact same metabolites was not performed in independent cohorts, therefore the performance of these profiles in other population warrants further investigation. Fifth, a comprehensive evaluation of model performance requires consideration of multiple indicators beyond the limited metrics used in this study. Future studies should also incorporate additional factors such as model interpretability, robustness, and generalizability to ensure effective model performance in real-world applications.

## Conclusions

This cohort study identified GCIPLT metabolic profiles that informed risks of mortality and 6 common diseases beyond clinical indicators in the general European population, and validated the hypothesis in a southern Chinese diabetic population by confirming the role of GCIPLT metabolic profiles in CVD risk stratification using a different metabolomic approach. Our evidence suggests that incorporating these profiles may assist in individualized risk stratification for risks of various health outcomes, contributing new insights into the retina as a unique window to systemic health.

## Notes

Supplement 1. **eFigure 1.** Pipeline of the Study **eFigure 2.** Analytic Framework of the Study **eFigure 3.** Heatmaps Demonstrating the Overall Correlations of GCIPLT and Metabolites **eFigure 4.** Associations of GCIPLT Metabolic Profiles and Risk of Morbidity of Common Diseases and Mortality **eFigure 5.** Cumulative Event Rates Over the Observation Time for Common Diseases and Mortality **eFigure 6.** Predictive Power of GCIPLT Metabolic Profiles and Clinical Indicators for Common Diseases and Mortality **eFigure 7.** Receiver Operating Characteristic Curves of Clinical Indicators-based Models, GCIPLT Metabolic State Models, and Combined Models for Predicting Common Diseases and Mortality **eFigure 8.** Calibration Plots Illustrating Predicted and Observed Probabilities for Common Diseases and Mortality **eFigure 9.** Net Benefit Curves of Clinical Utility for Common Diseases and Mortality **eTable 1.** List Summarizing All Metabolic Markers Quantifying Using 1H-NMR Profiling **eTable 2.** Metabolic Markers Used in Models Discriminating Common Diseases and Mortality **eTable 3.** Baseline Characteristics of the Study Population from the UKB Cohort **eTable 4.** Baseline Characteristics of the Study Population from the GDES Cohort **eTable 5.** Significant Metabolites Associated With GCIPLT **eTable 6.** Number of Incident Health Outcomes in Total, Discovery Set, and Validation Set **eTable 7.** Discriminative Power of Clinical Indicators and GCIPLT Metabolic Profiles for Predicting Mortality and Common Diseases **eTable 8.** NRIs Improvements of Incorporating GCIPLT Metabolic Profiles for Mortality and Morbidity of Common Diseases **eTable 9.** List Summarizing Metabolic Profiles Identified in the GDES Cohort Using LC/MS Profiling **eTable 10.** Sensitivity Analysis of Excluding All Missing Values
eMethods.
**eAppendix.** Extended Discussion

Supplement 2.
Data Sharing Statement
