# Supplementary material for: Analysis of Plasma Metabolic Profile on Ganglion Cell–Inner Plexiform Layer Thickness With Mortality and Common Diseases
Source: JAMA Netw Open. 2023 May 16;6(5):e2313220. doi: 10.1001/jamanetworkopen.2023.13220 (PMC10189567; doi:10.1001/jamanetworkopen.2023.13220)
Supplement: Supplement 1. — eFigure 1. Pipeline of the Study eFigure 2. Analytic Framework of the Study eFigure 3. Heatmaps Demonstrating the Overall Correlations of GCIPLT and Metabolites eFigure 4. Associations of GCIPLT Metabolic Profiles and Risk of Morbidity of Common Diseases and Mortality eFigure 5. Cumulative Event Rates Over the Observation Time for Common Diseases and Mortality eFigure 6. Predictive Power of GCIPLT Metabolic Profiles and Clinical Indicators for Common Diseases and Mortality eFigure 7. Receiver Operating Characteristic Curves of Clinical Indicators-based Models, GCIPLT Metabolic State Models, and Combined Models for Predicting Common Diseases and Mortality eFigure 8. Calibration Plots Illustrating Predicted and Observed Probabilities for Common Diseases and Mortality eFigure 9. Net Benefit Curves of Clinical Utility for Common Diseases and Mortality eTable 1. List Summarizing All Metabolic Markers Quantifying Using 1H-NMR Profiling eTable 2. Metabolic Markers Used in Models Discriminating Common Diseases and Mortality eTable 3. Baseline Characteristics of the Study Population from the UKB Cohort eTable 4. Baseline Characteristics of the Study Population from the GDES Cohort eTable 5. Significant Metabolites Associated With GCIPLT eTable 6. Number of Incident Health Outcomes in Total, Discovery Set, and Validation Set eTable 7. Discriminative Power of Clinical Indicators and GCIPLT Metabolic Profiles for Predicting Mortality and Common Diseases eTable 8. NRIs Improvements of Incorporating GCIPLT Metabolic Profiles for Mortality and Morbidity of Common Diseases eTable 9. List Summarizing Metabolic Profiles Identified in the GDES Cohort Using LC/MS Profiling eTable 10. Sensitivity Analysis of Excluding All Missing Values eMethods. eAppendix. Extended Discussion [file jamanetwopen-e2313220-s001.pdf]

Supplemental Online Content

Yang S, Zhu Z, Yuan Y, et al. Analysis of plasma metabolic profiles on ganglion cell–inner plexiform layer thickness with mortality and common diseases. *JAMA Netw Open.* 2023;6(5):e2313220.
doi:10.1001/jamanetworkopen.2023.13220

eFigure 1. Pipeline of the Study

eFigure 2. Analytic Framework of the Study

eFigure 3. Heatmaps Demonstrating the Overall Correlations of GCIPLT and Metabolites

eFigure 4. Associations of GCIPLT Metabolic Profiles and Risk of Morbidity of Common Diseases and Mortality

eFigure 5. Cumulative Event Rates Over the Observation Time for Common Diseases and Mortality

eFigure 6. Predictive Power of GCIPLT Metabolic Profiles and Clinical Indicators for Common Diseases and Mortality

eFigure 7. Receiver Operating Characteristic Curves of Clinical Indicators-based Models, GCIPLT Metabolic State Models, and Combined Models for Predicting Common Diseases and Mortality

eFigure 8. Calibration Plots Illustrating Predicted and Observed Probabilities for Common Diseases and Mortality

eFigure 9. Net Benefit Curves of Clinical Utility for Common Diseases and Mortality

eTable 1. List Summarizing All Metabolic Markers Quantifying Using 1H-NMR Profiling

eTable 2. Metabolic Markers Used in Models Discriminating Common Diseases and Mortality

eTable 3. Baseline Characteristics of the Study Population from the UKB Cohort

eTable 4. Baseline Characteristics of the Study Population from the GDES Cohort

eTable 5. Significant Metabolites Associated With GCIPLT

eTable 6. Number of Incident Health Outcomes in Total, Discovery Set, and Validation Set

eTable 7. Discriminative Power of Clinical Indicators and GCIPLT Metabolic Profiles for Predicting Mortality and Common Diseases

© 2023 Yang S et al. *JAMA Network Open.*

eTable 8. NRIs Improvements of Incorporating GCIPLT Metabolic Profiles for Mortality and Morbidity of Common Diseases

eTable 9. List Summarizing Metabolic Profiles Identified in the GDES Cohort Using LC/MS Profiling

eTable 10. Sensitivity Analysis of Excluding All Missing Values

eMethods.

eAppendix. Extended Discussion

This supplemental material has been provided by the authors to give readers additional information about their work.

© 2023 Yang S et al. *JAMA Network Open.*

eFigure 1. Pipeline of the study.

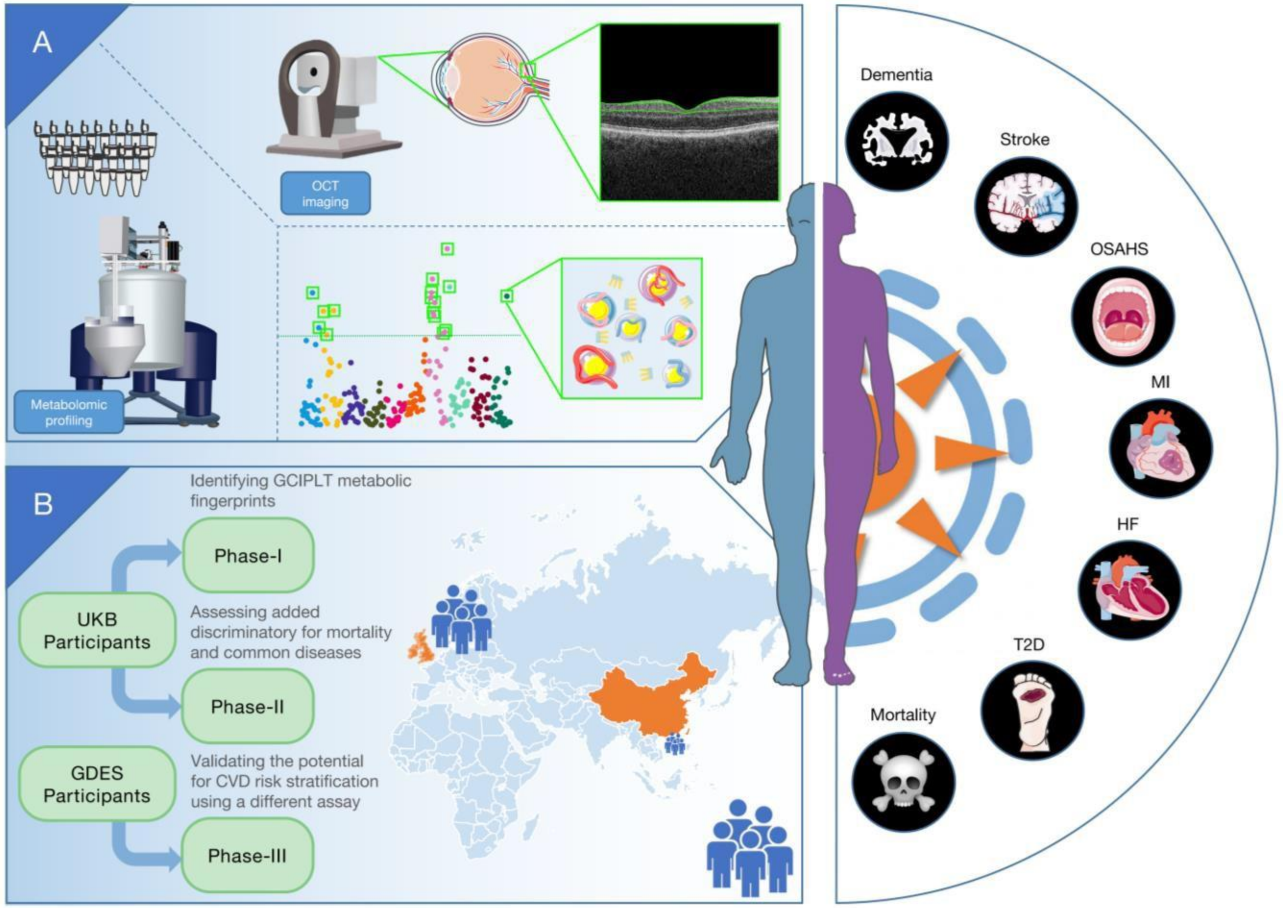

© 2023 Yang S et al. *JAMA Network Open.*

eFigure 2.

The analytic framework of the study.

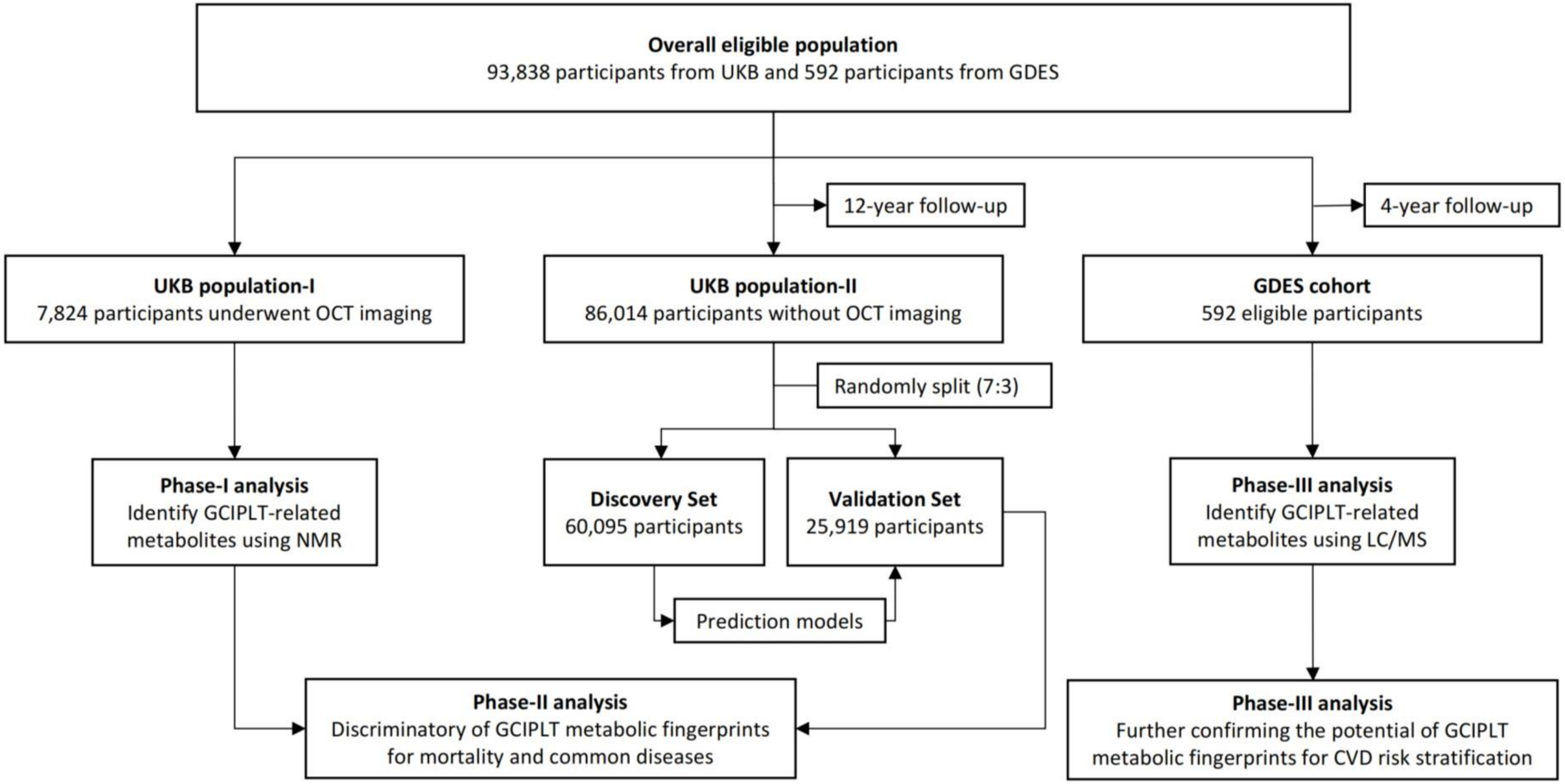

© 2023 Yang S et al. *JAMA Network Open.*

eFigure 3.

Heatmaps demonstrating the overall correlations of GCIPLT and metabolites.

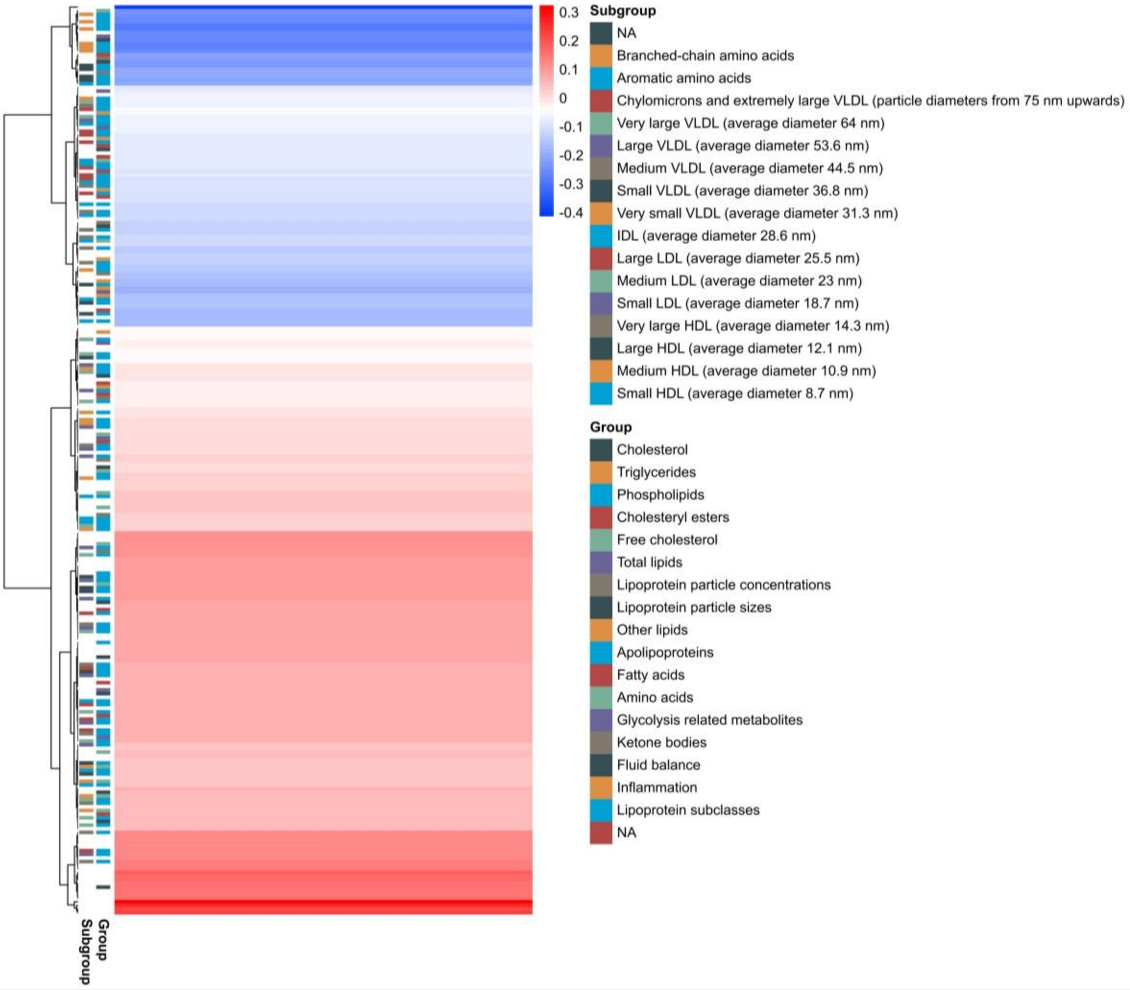

© 2023 Yang S et al. *JAMA Network Open.*

eFigure 4.

Associations of GCIPLT metabolic profiles and risk of morbidity of common diseases and mortality.

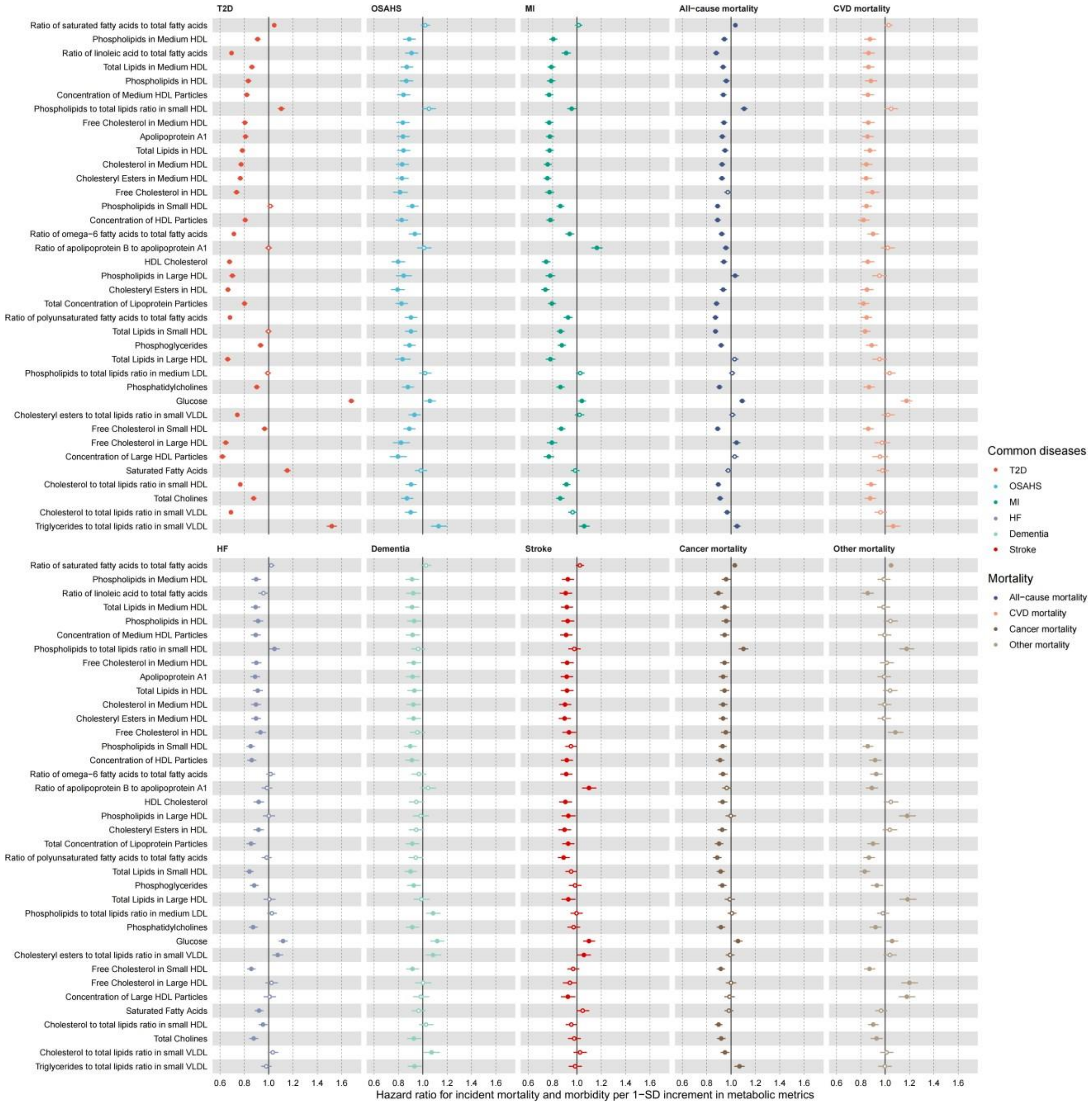

© 2023 Yang S et al. *JAMA Network Open.*

eFigure 5.

Cumulative event rates over the observation time for common diseases and mortality.

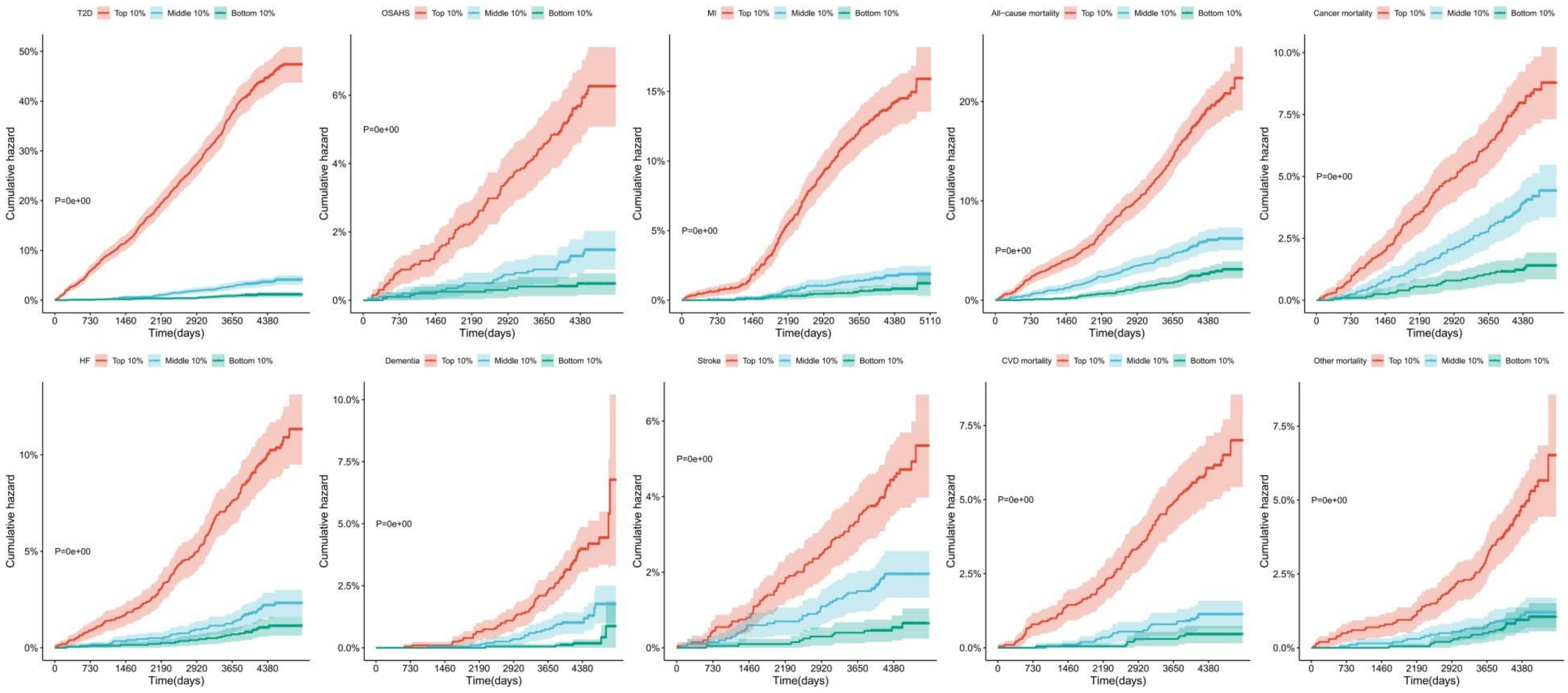

© 2023 Yang S et al. *JAMA Network Open.*

eFigure 6.

Predictive power of GCIPLT metabolic profiles and clinical indicators for common diseases and mortality.

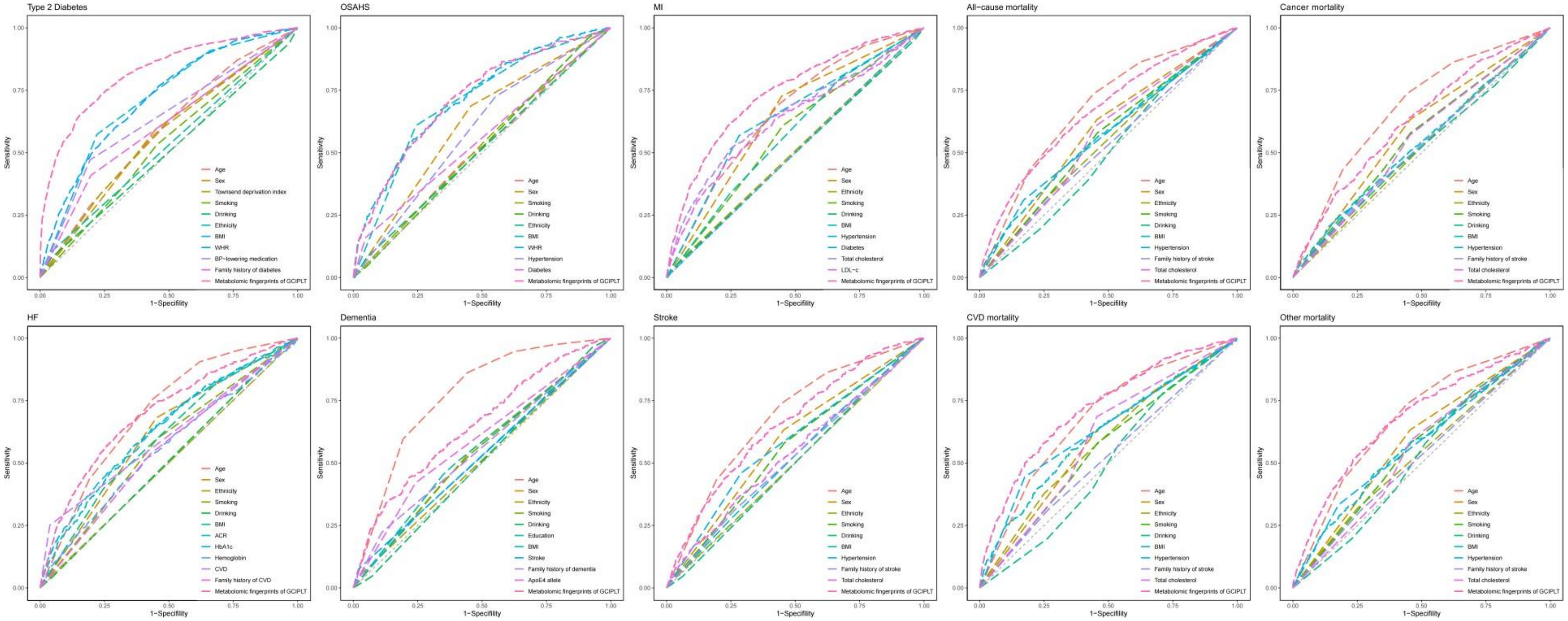

© 2023 Yang S et al. *JAMA Network Open.*

eFigure 7

Receiver operating characteristic curves of clinical indicators-based models, GCIPLT metabolic state models, and combined models for predicting common diseases and mortality.

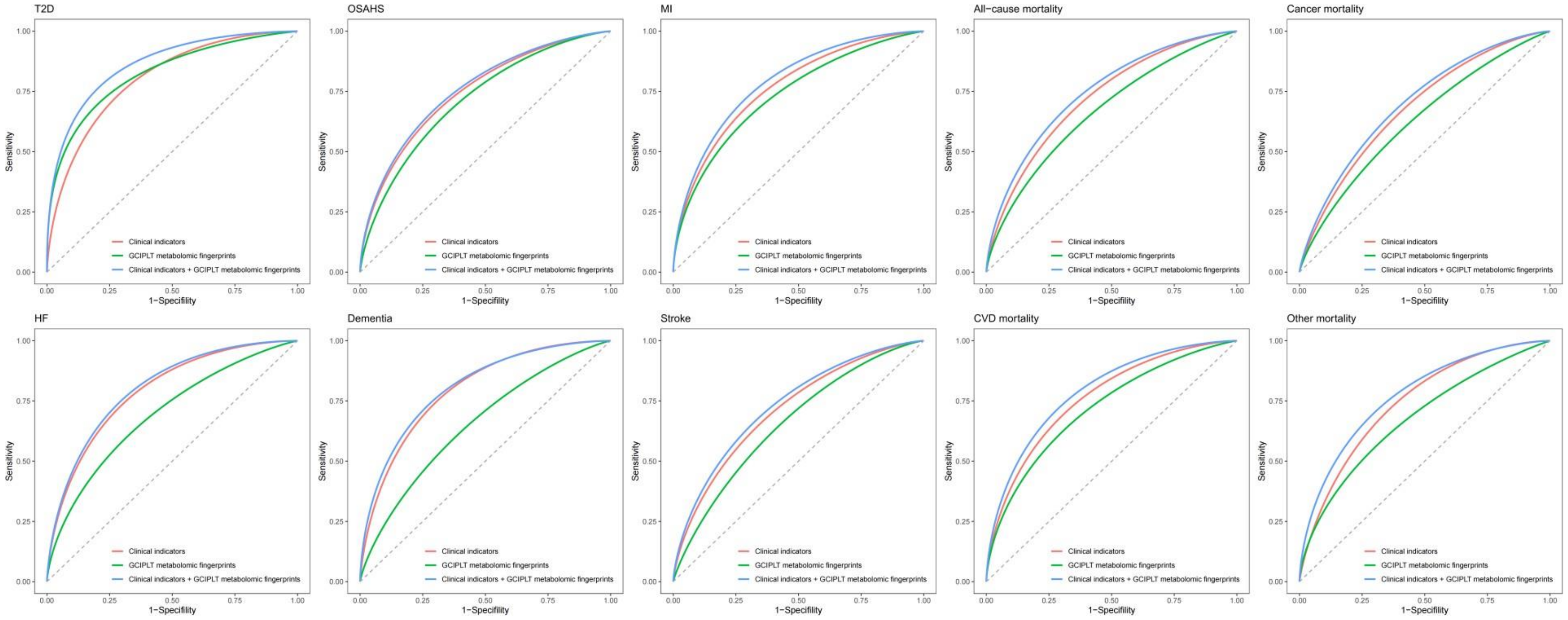

© 2023 Yang S et al. *JAMA Network Open.*

eFigure 8

Calibration plots illustrating predicted and observed probabilities for common diseases and mortality.

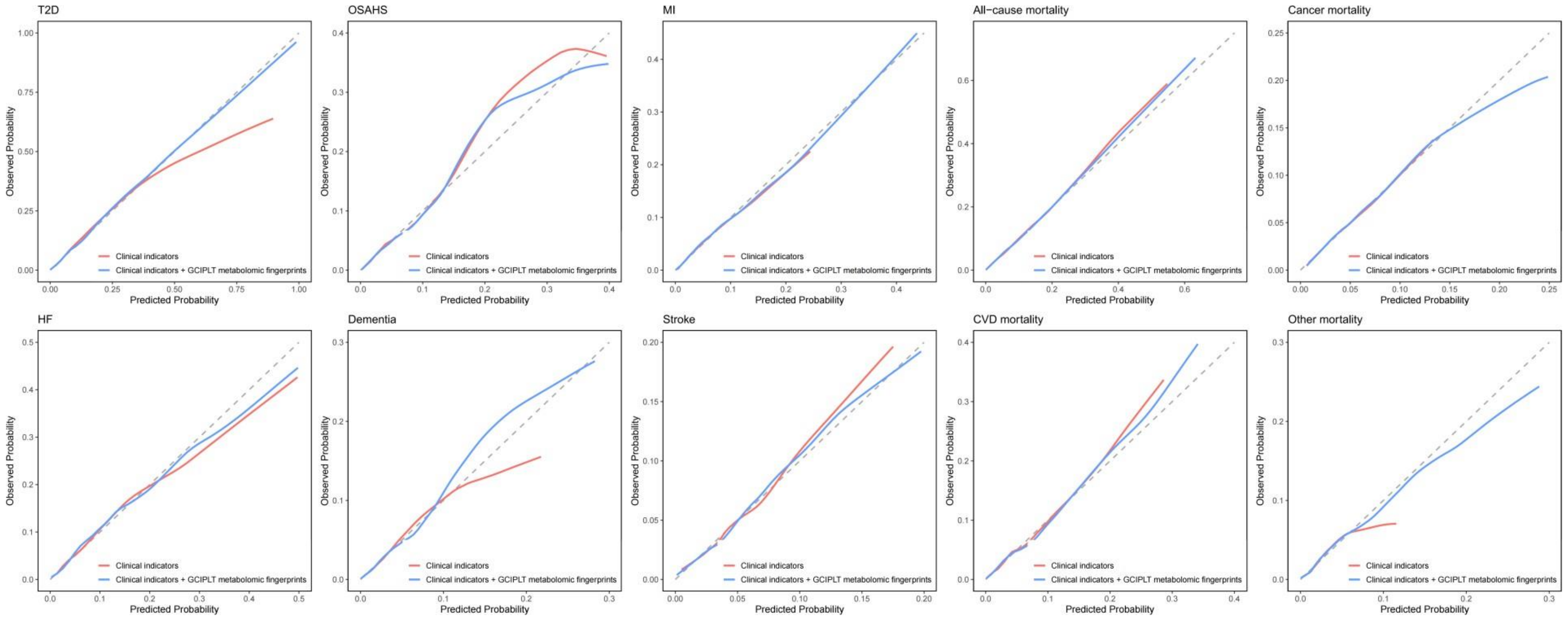

© 2023 Yang S et al. *JAMA Network Open.*

eFigure 9

Net benefit curves of clinical utility for common diseases and mortality.

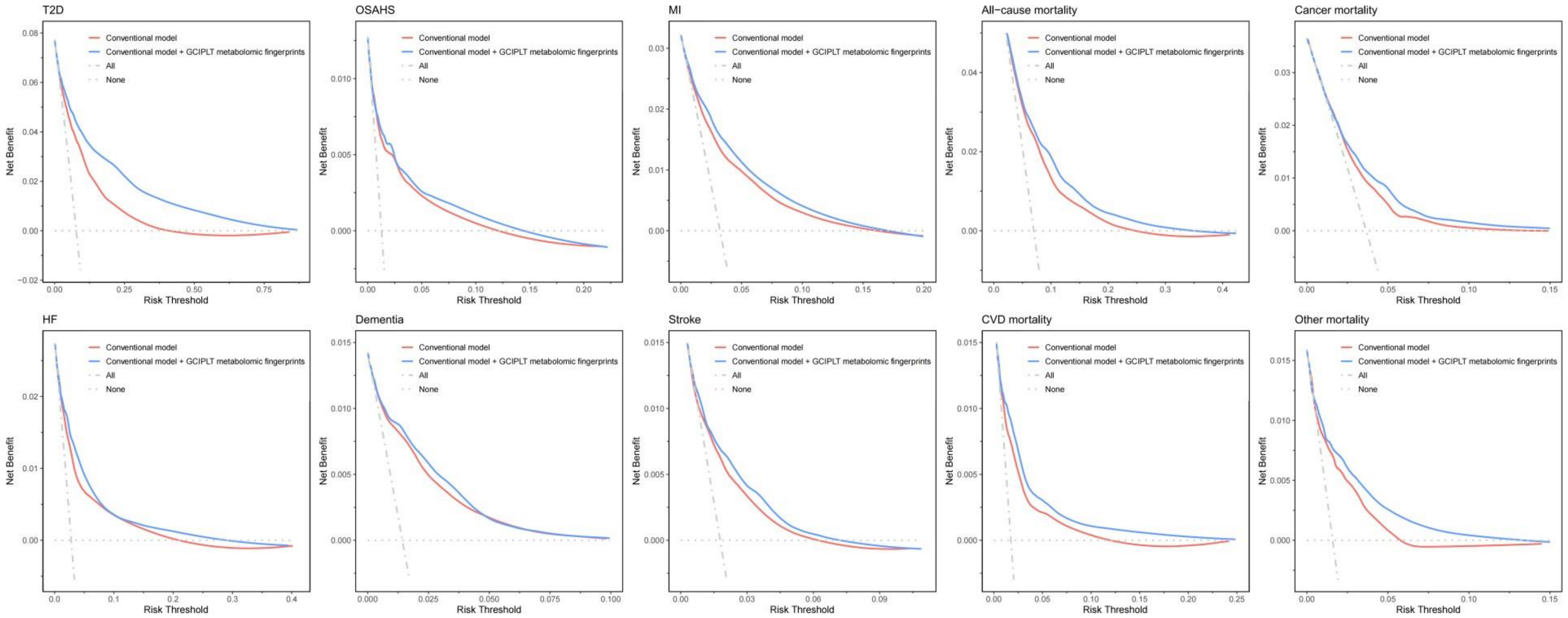

© 2023 Yang S et al. *JAMA Network Open.*

eTable 1

List summarizing all metabolic markers quantifying using 1H-NMR profiling.

**Metabolic metrics**	**Units**	**Group**	**Subgroup**
Total Cholesterol	mmol/l	Cholesterol	N/A
Total Cholesterol Minus HDL-C	mmol/l	Cholesterol	N/A
Remnant Cholesterol (Non-HDL, Non-LDL -Cholesterol)	mmol/l	Cholesterol	N/A
VLDL Cholesterol	mmol/l	Cholesterol	N/A
Clinical LDL Cholesterol	mmol/l	Cholesterol	N/A
LDL Cholesterol	mmol/l	Cholesterol	N/A
HDL Cholesterol	mmol/l	Cholesterol	N/A
Total Triglycerides	mmol/l	Triglycerides	N/A
Triglycerides in VLDL	mmol/l	Triglycerides	N/A
Triglycerides in LDL	mmol/l	Triglycerides	N/A
Triglycerides in HDL	mmol/l	Triglycerides	N/A
Total Phospholipids in Lipoprotein Particles	mmol/l	Phospholipids	N/A
Phospholipids in VLDL	mmol/l	Phospholipids	N/A
Phospholipids in LDL	mmol/l	Phospholipids	N/A
Phospholipids in HDL	mmol/l	Phospholipids	N/A
Total Esterified Cholesterol	mmol/l	Cholesteryl esters	N/A
Cholesteryl Esters in VLDL	mmol/l	Cholesteryl esters	N/A
Cholesteryl Esters in LDL	mmol/l	Cholesteryl esters	N/A
Cholesteryl Esters in HDL	mmol/l	Cholesteryl esters	N/A
Total Free Cholesterol	mmol/l	Free cholesterol	N/A
Free Cholesterol in VLDL	mmol/l	Free cholesterol	N/A
Free Cholesterol in LDL	mmol/l	Free cholesterol	N/A
Free Cholesterol in HDL	mmol/l	Free cholesterol	N/A
Total Lipids in Lipoprotein Particles	mmol/l	Total lipids	N/A
Total Lipids in VLDL	mmol/l	Total lipids	N/A
Total Lipids in LDL	mmol/l	Total lipids	N/A
Total Lipids in HDL	mmol/l	Total lipids	N/A
Total Concentration of Lipoprotein Particles	mmol/l	Lipoprotein particle concentrations	N/A
Concentration of VLDL Particles	mmol/l	Lipoprotein particle concentrations	N/A
Concentration of LDL Particles	mmol/l	Lipoprotein	N/A

© 2023 Yang S et al. *JAMA Network Open.*

		particle concentrations	
Concentration of HDL Particles	mmol/l	Lipoprotein particle concentrations	N/A
Average Diameter for VLDL Particles	nm	Lipoprotein particle sizes	N/A
Average Diameter for LDL Particles	nm	Lipoprotein particle sizes	N/A
Average Diameter for HDL Particles	nm	Lipoprotein particle sizes	N/A
Phosphoglycerides	mmol/l	Other lipids	N/A
Total Cholines	mmol/l	Other lipids	N/A
Phosphatidylcholines	mmol/l	Other lipids	N/A
Sphingomyelins	mmol/l	Other lipids	N/A
Apolipoprotein B	g/l	Apolipoproteins	N/A
Apolipoprotein A1	g/l	Apolipoproteins	N/A
Total Fatty Acids	mmol/l	Fatty acids	N/A
Degree of Unsaturation	degree	Fatty acids	N/A
Omega-3 Fatty Acids	mmol/l	Fatty acids	N/A
Omega-6 Fatty Acids	mmol/l	Fatty acids	N/A
Polyunsaturated Fatty Acids	mmol/l	Fatty acids	N/A
Monounsaturated Fatty Acids	mmol/l	Fatty acids	N/A
Saturated Fatty Acids	mmol/l	Fatty acids	N/A
Linoleic Acid	mmol/l	Fatty acids	N/A
Docosahexaenoic Acid	mmol/l	Fatty acids	N/A
Alanine	mmol/l	Amino acids	N/A
Glutamine	mmol/l	Amino acids	N/A
Glycine	mmol/l	Amino acids	N/A
Histidine	mmol/l	Amino acids	N/A
Total Concentration of Branched-Chain Amino Acids (Leucine + Isoleucine + Valine)	mmol/l	Amino acids	Branched-chain amino acids
Isoleucine	mmol/l	Amino acids	Branched-chain amino acids
Leucine	mmol/l	Amino acids	Branched-chain amino acids
Valine	mmol/l	Amino acids	Branched-chain amino acids
Phenylalanine	mmol/l	Amino acids	Aromatic amino acids
Tyrosine	mmol/l	Amino acids	Aromatic amino acids
Glucose	mmol/l	Glycolysis related metabolites	N/A

© 2023 Yang S et al. *JAMA Network Open.*

Lactate	mmol/l	Glycolysis related metabolites	N/A
Pyruvate	mmol/l	Glycolysis related metabolites	N/A
Citrate	mmol/l	Glycolysis related metabolites	N/A
Acetate	mmol/l	Ketone bodies	N/A
Acetoacetate	mmol/l	Ketone bodies	N/A
Acetone	mmol/l	Ketone bodies	N/A
Creatinine	mmol/l	Ketone bodies	N/A
Albumin	mmol/l	Fluid balance	N/A
Glycoprotein Acetyls	g/l	Fluid balance	N/A
Concentration of Chylomicrons and Extremely Large VLDL Particles	mmol/l	Inflammation	N/A
Total Lipids in Chylomicrons and Extremely Large VLDL	mmol/l	Lipoprotein subclasses	Chylomicrons and extremely large VLDL (particle diameters from 75 nm upwards)
Phospholipids in Chylomicrons and Extremely Large VLDL	mmol/l	Lipoprotein subclasses	Chylomicrons and extremely large VLDL (particle diameters from 75 nm upwards)
Cholesterol in Chylomicrons and Extremely Large VLDL	mmol/l	Lipoprotein subclasses	Chylomicrons and extremely large VLDL (particle diameters from 75 nm upwards)
Cholesteryl Esters in Chylomicrons and Extremely Large VLDL	mmol/l	Lipoprotein subclasses	Chylomicrons and extremely large VLDL (particle diameters from 75 nm upwards)
Free Cholesterol in Chylomicrons and Extremely Large VLDL	mmol/l	Lipoprotein subclasses	Chylomicrons and extremely large VLDL (particle diameters from 75 nm upwards)
Triglycerides in Chylomicrons and Extremely Large VLDL	mmol/l	Lipoprotein subclasses	Chylomicrons and extremely large VLDL (particle diameters from 75 nm upwards)
Concentration of Very Large VLDL Particles	mmol/l	Lipoprotein subclasses	Chylomicrons and extremely large VLDL (particle diameters from 75 nm upwards)
Total Lipids in Very Large VLDL	mmol/l	Lipoprotein	Very large VLDL (average

© 2023 Yang S et al. *JAMA Network Open.*

		subclasses	diameter 64 nm)
Phospholipids in Very Large VLDL	mmol/l	Lipoprotein subclasses	Very large VLDL (average diameter 64 nm)
Cholesterol in Very Large VLDL	mmol/l	Lipoprotein subclasses	Very large VLDL (average diameter 64 nm)
Cholesteryl Esters in Very Large VLDL	mmol/l	Lipoprotein subclasses	Very large VLDL (average diameter 64 nm)
Free Cholesterol in Very Large VLDL	mmol/l	Lipoprotein subclasses	Very large VLDL (average diameter 64 nm)
Triglycerides in Very Large VLDL	mmol/l	Lipoprotein subclasses	Very large VLDL (average diameter 64 nm)
Concentration of Large VLDL Particles	mmol/l	Lipoprotein subclasses	Very large VLDL (average diameter 64 nm)
Total Lipids in Large VLDL	mmol/l	Lipoprotein subclasses	Large VLDL (average diameter 53.6 nm)
Phospholipids in Large VLDL	mmol/l	Lipoprotein subclasses	Large VLDL (average diameter 53.6 nm)
Cholesterol in Large VLDL	mmol/l	Lipoprotein subclasses	Large VLDL (average diameter 53.6 nm)
Cholesteryl Esters in Large VLDL	mmol/l	Lipoprotein subclasses	Large VLDL (average diameter 53.6 nm)
Free Cholesterol in Large VLDL	mmol/l	Lipoprotein subclasses	Large VLDL (average diameter 53.6 nm)
Triglycerides in Large VLDL	mmol/l	Lipoprotein subclasses	Large VLDL (average diameter 53.6 nm)
Concentration of Medium VLDL Particles	mmol/l	Lipoprotein subclasses	Large VLDL (average diameter 53.6 nm)
Total Lipids in Medium VLDL	mmol/l	Lipoprotein subclasses	Medium VLDL (average diameter 44.5 nm)
Phospholipids in Medium VLDL	mmol/l	Lipoprotein subclasses	Medium VLDL (average diameter 44.5 nm)
Cholesterol in Medium VLDL	mmol/l	Lipoprotein subclasses	Medium VLDL (average diameter 44.5 nm)
Cholesteryl Esters in Medium VLDL	mmol/l	Lipoprotein subclasses	Medium VLDL (average diameter 44.5 nm)
Free Cholesterol in Medium VLDL	mmol/l	Lipoprotein subclasses	Medium VLDL (average diameter 44.5 nm)
Triglycerides in Medium VLDL	mmol/l	Lipoprotein subclasses	Medium VLDL (average diameter 44.5 nm)
Concentration of Small VLDL Particles	mmol/l	Lipoprotein subclasses	Medium VLDL (average diameter 44.5 nm)
Total Lipids in Small VLDL	mmol/l	Lipoprotein subclasses	Small VLDL (average diameter 36.8 nm)

© 2023 Yang S et al. *JAMA Network Open.*

Phospholipids in Small VLDL	mmol/l	Lipoprotein subclasses	Small VLDL (average diameter 36.8 nm)
Cholesterol in Small VLDL	mmol/l	Lipoprotein subclasses	Small VLDL (average diameter 36.8 nm)
Cholesteryl Esters in Small VLDL	mmol/l	Lipoprotein subclasses	Small VLDL (average diameter 36.8 nm)
Free Cholesterol in Small VLDL	mmol/l	Lipoprotein subclasses	Small VLDL (average diameter 36.8 nm)
Triglycerides in Small VLDL	mmol/l	Lipoprotein subclasses	Small VLDL (average diameter 36.8 nm)
Concentration of Very Small VLDL Particles	mmol/l	Lipoprotein subclasses	Small VLDL (average diameter 36.8 nm)
Total Lipids in Very Small VLDL	mmol/l	Lipoprotein subclasses	Very small VLDL (average diameter 31.3 nm)
Phospholipids in Very Small VLDL	mmol/l	Lipoprotein subclasses	Very small VLDL (average diameter 31.3 nm)
Cholesterol in Very Small VLDL	mmol/l	Lipoprotein subclasses	Very small VLDL (average diameter 31.3 nm)
Cholesteryl Esters in Very Small VLDL	mmol/l	Lipoprotein subclasses	Very small VLDL (average diameter 31.3 nm)
Free Cholesterol in Very Small VLDL	mmol/l	Lipoprotein subclasses	Very small VLDL (average diameter 31.3 nm)
Triglycerides in Very Small VLDL	mmol/l	Lipoprotein subclasses	Very small VLDL (average diameter 31.3 nm)
Concentration of IDL Particles	mmol/l	Lipoprotein subclasses	Very small VLDL (average diameter 31.3 nm)
Total Lipids in IDL	mmol/l	Lipoprotein subclasses	IDL (average diameter 28.6 nm)
Phospholipids in IDL	mmol/l	Lipoprotein subclasses	IDL (average diameter 28.6 nm)
Cholesterol in IDL	mmol/l	Lipoprotein subclasses	IDL (average diameter 28.6 nm)
Cholesteryl Esters in IDL	mmol/l	Lipoprotein subclasses	IDL (average diameter 28.6 nm)
Free Cholesterol in IDL	mmol/l	Lipoprotein subclasses	IDL (average diameter 28.6 nm)
Triglycerides in IDL	mmol/l	Lipoprotein subclasses	IDL (average diameter 28.6 nm)
Concentration of Large LDL Particles	mmol/l	Lipoprotein subclasses	IDL (average diameter 28.6 nm)
Total Lipids in Large LDL	mmol/l	Lipoprotein subclasses	Large LDL (average diameter 25.5 nm)
Phospholipids in Large LDL	mmol/l	Lipoprotein subclasses	Large LDL (average diameter 25.5 nm)

© 2023 Yang S et al. *JAMA Network Open.*

Cholesterol in Large LDL	mmol/l	Lipoprotein subclasses	Large LDL (average diameter 25.5 nm)
Cholesteryl Esters in Large LDL	mmol/l	Lipoprotein subclasses	Large LDL (average diameter 25.5 nm)
Free Cholesterol in Large LDL	mmol/l	Lipoprotein subclasses	Large LDL (average diameter 25.5 nm)
Triglycerides in Large LDL	mmol/l	Lipoprotein subclasses	Large LDL (average diameter 25.5 nm)
Concentration of Medium LDL Particles	mmol/l	Lipoprotein subclasses	Large LDL (average diameter 25.5 nm)
Total Lipids in Medium LDL	mmol/l	Lipoprotein subclasses	Medium LDL (average diameter 23 nm)
Phospholipids in Medium LDL	mmol/l	Lipoprotein subclasses	Medium LDL (average diameter 23 nm)
Cholesterol in Medium LDL	mmol/l	Lipoprotein subclasses	Medium LDL (average diameter 23 nm)
Cholesteryl Esters in Medium LDL	mmol/l	Lipoprotein subclasses	Medium LDL (average diameter 23 nm)
Free Cholesterol in Medium LDL	mmol/l	Lipoprotein subclasses	Medium LDL (average diameter 23 nm)
Triglycerides in Medium LDL	mmol/l	Lipoprotein subclasses	Medium LDL (average diameter 23 nm)
Concentration of Small LDL Particles	mmol/l	Lipoprotein subclasses	Medium LDL (average diameter 23 nm)
Total Lipids in Small LDL	mmol/l	Lipoprotein subclasses	Small LDL (average diameter 18.7 nm)
Phospholipids in Small LDL	mmol/l	Lipoprotein subclasses	Small LDL (average diameter 18.7 nm)
Cholesterol in Small LDL	mmol/l	Lipoprotein subclasses	Small LDL (average diameter 18.7 nm)
Cholesteryl Esters in Small LDL	mmol/l	Lipoprotein subclasses	Small LDL (average diameter 18.7 nm)
Free Cholesterol in Small LDL	mmol/l	Lipoprotein subclasses	Small LDL (average diameter 18.7 nm)
Triglycerides in Small LDL	mmol/l	Lipoprotein subclasses	Small LDL (average diameter 18.7 nm)
Concentration of Very Large HDL Particles	mmol/l	Lipoprotein subclasses	Small LDL (average diameter 18.7 nm)
Total Lipids in Very Large HDL	mmol/l	Lipoprotein subclasses	Very large HDL (average diameter 14.3 nm)
Phospholipids in Very Large HDL	mmol/l	Lipoprotein subclasses	Very large HDL (average diameter 14.3 nm)
Cholesterol in Very Large HDL	mmol/l	Lipoprotein subclasses	Very large HDL (average diameter 14.3 nm)

© 2023 Yang S et al. *JAMA Network Open.*

Cholesteryl Esters in Very Large HDL	mmol/l	Lipoprotein subclasses	Very large HDL (average diameter 14.3 nm)
Free Cholesterol in Very Large HDL	mmol/l	Lipoprotein subclasses	Very large HDL (average diameter 14.3 nm)
Triglycerides in Very Large HDL	mmol/l	Lipoprotein subclasses	Very large HDL (average diameter 14.3 nm)
Concentration of Large HDL Particles	mmol/l	Lipoprotein subclasses	Very large HDL (average diameter 14.3 nm)
Total Lipids in Large HDL	mmol/l	Lipoprotein subclasses	Large HDL (average diameter 12.1 nm)
Phospholipids in Large HDL	mmol/l	Lipoprotein subclasses	Large HDL (average diameter 12.1 nm)
Cholesterol in Large HDL	mmol/l	Lipoprotein subclasses	Large HDL (average diameter 12.1 nm)
Cholesteryl Esters in Large HDL	mmol/l	Lipoprotein subclasses	Large HDL (average diameter 12.1 nm)
Free Cholesterol in Large HDL	mmol/l	Lipoprotein subclasses	Large HDL (average diameter 12.1 nm)
Triglycerides in Large HDL	mmol/l	Lipoprotein subclasses	Large HDL (average diameter 12.1 nm)
Concentration of Medium HDL Particles	mmol/l	Lipoprotein subclasses	Large HDL (average diameter 12.1 nm)
Total Lipids in Medium HDL	mmol/l	Lipoprotein subclasses	Medium HDL (average diameter 10.9 nm)
Phospholipids in Medium HDL	mmol/l	Lipoprotein subclasses	Medium HDL (average diameter 10.9 nm)
Cholesterol in Medium HDL	mmol/l	Lipoprotein subclasses	Medium HDL (average diameter 10.9 nm)
Cholesteryl Esters in Medium HDL	mmol/l	Lipoprotein subclasses	Medium HDL (average diameter 10.9 nm)
Free Cholesterol in Medium HDL	mmol/l	Lipoprotein subclasses	Medium HDL (average diameter 10.9 nm)
Triglycerides in Medium HDL	mmol/l	Lipoprotein subclasses	Medium HDL (average diameter 10.9 nm)
Concentration of Small HDL Particles	mmol/l	Lipoprotein subclasses	Medium HDL (average diameter 10.9 nm)
Total Lipids in Small HDL	mmol/l	Lipoprotein subclasses	Small HDL (average diameter 8.7 nm)
Phospholipids in Small HDL	mmol/l	Lipoprotein subclasses	Small HDL (average diameter 8.7 nm)
Cholesterol in Small HDL	mmol/l	Lipoprotein subclasses	Small HDL (average diameter 8.7 nm)
Cholesteryl Esters in Small HDL	mmol/l	Lipoprotein subclasses	Small HDL (average diameter 8.7 nm)

© 2023 Yang S et al. *JAMA Network Open.*

Free Cholesterol in Small HDL	mmol/l	Lipoprotein subclasses	Small HDL (average diameter 8.7 nm)
Triglycerides in Small HDL	mmol/l	Lipoprotein subclasses	Small HDL (average diameter 8.7 nm)
3-Hydroxybutyrate	mmol/l	Ketone bodies	N/A
Ratio of triglycerides to phosphoglycerides	N/A	Ratios	N/A
Ratio of apolipoprotein B to apolipoprotein A1	N/A	Ratios	N/A
Ratio of omega-3 fatty acids to total fatty acids	N/A	Ratios	N/A
Ratio of omega-6 fatty acids to total fatty acids	N/A	Ratios	N/A
Ratio of polyunsaturated fatty acids to total fatty acids	N/A	Ratios	N/A
Ratio of monounsaturated fatty acids to total fatty acids	N/A	Ratios	N/A
Ratio of saturated fatty acids to total fatty acids	N/A	Ratios	N/A
Ratio of linoleic acid to total fatty acids	N/A	Ratios	N/A
Ratio of docosahexaenoic acid to total fatty acids	N/A	Ratios	N/A
Ratio of polyunsaturated fatty acids to monounsaturated fatty acids	N/A	Ratios	N/A
Ratio of omega-6 fatty acids to omega-3 fatty acids	N/A	Ratios	N/A
Phospholipids to total lipids ratio in chylomicrons and extremely large VLDL	N/A	Ratios	N/A
Cholesterol to total lipids ratio in chylomicrons and extremely large VLDL	N/A	Ratios	N/A
Cholesteryl esters to total lipids ratio in chylomicrons and extremely large VLD	N/A	Ratios	N/A
Free cholesterol to total lipids ratio in chylomicrons and extremely large VLDL	N/A	Ratios	N/A
Triglycerides to total lipids ratio in chylomicrons and extremely large VLDL	N/A	Ratios	N/A
Phospholipids to total lipids	N/A	Ratios	N/A

© 2023 Yang S et al. *JAMA Network Open.*

ratio in very large VLDL			
Cholesterol to total lipids ratio in very large VLDL	N/A	Ratios	N/A
Cholesteryl esters to total lipids ratio in very large VLDL	N/A	Ratios	N/A
Free cholesterol to total lipids ratio in very large VLDL	N/A	Ratios	N/A
Triglycerides to total lipids ratio in very large VLDL	N/A	Ratios	N/A
Phospholipids to total lipids ratio in large VLDL	N/A	Ratios	N/A
Cholesterol to total lipids ratio in large VLDL	N/A	Ratios	N/A
Cholesteryl esters to total lipids ratio in large VLDL	N/A	Ratios	N/A
Free cholesterol to total lipids ratio in large VLDL	N/A	Ratios	N/A
Triglycerides to total lipids ratio in large VLDL	N/A	Ratios	N/A
Phospholipids to total lipids ratio in medium VLDL	N/A	Ratios	N/A
Cholesterol to total lipids ratio in medium VLDL	N/A	Ratios	N/A
Cholesteryl esters to total lipids ratio in medium VLDL	N/A	Ratios	N/A
Free cholesterol to total lipids ratio in medium VLDL	N/A	Ratios	N/A
Triglycerides to total lipids ratio in medium VLDL	N/A	Ratios	N/A
Phospholipids to total lipids ratio in small VLDL	N/A	Ratios	N/A
Cholesterol to total lipids ratio in small VLDL	N/A	Ratios	N/A
Cholesteryl esters to total lipids ratio in small VLDL	N/A	Ratios	N/A
Free cholesterol to total lipids ratio in small VLDL	N/A	Ratios	N/A
Triglycerides to total lipids ratio in small VLDL	N/A	Ratios	N/A
Phospholipids to total lipids ratio in very small VLDL	N/A	Ratios	N/A
Cholesterol to total lipids ratio in very small VLDL	N/A	Ratios	N/A

© 2023 Yang S et al. *JAMA Network Open.*

Cholesteryl esters to total lipids ratio in very small VLDL	N/A	Ratios	N/A
Free cholesterol to total lipids ratio in very small VLDL	N/A	Ratios	N/A
Triglycerides to total lipids ratio in very small VLDL	N/A	Ratios	N/A
Phospholipids to total lipids ratio in IDL	N/A	Ratios	N/A
Cholesterol to total lipids ratio in IDL	N/A	Ratios	N/A
Cholesteryl esters to total lipids ratio in IDL	N/A	Ratios	N/A
Free cholesterol to total lipids ratio in IDL	N/A	Ratios	N/A
Triglycerides to total lipids ratio in IDL	N/A	Ratios	N/A
Phospholipids to total lipids ratio in large LDL	N/A	Ratios	N/A
Cholesterol to total lipids ratio in large LDL	N/A	Ratios	N/A
Cholesteryl esters to total lipids ratio in large LDL	N/A	Ratios	N/A
Free cholesterol to total lipids ratio in large LDL	N/A	Ratios	N/A
Triglycerides to total lipids ratio in large LDL	N/A	Ratios	N/A
Phospholipids to total lipids ratio in medium LDL	N/A	Ratios	N/A
Cholesterol to total lipids ratio in medium LDL	N/A	Ratios	N/A
Cholesteryl esters to total lipids ratio in medium LDL	N/A	Ratios	N/A
Free cholesterol to total lipids ratio in medium LDL	N/A	Ratios	N/A
Triglycerides to total lipids ratio in medium LDL	N/A	Ratios	N/A
Phospholipids to total lipids ratio in small LDL	N/A	Ratios	N/A
Cholesterol to total lipids ratio in small LDL	N/A	Ratios	N/A
Cholesteryl esters to total lipids ratio in small LDL	N/A	Ratios	N/A
Free cholesterol to total lipids ratio in small LDL	N/A	Ratios	N/A

© 2023 Yang S et al. *JAMA Network Open.*

Triglycerides to total lipids ratio in small LDL	N/A	Ratios	N/A
Phospholipids to total lipids ratio in very large HDL	N/A	Ratios	N/A
Cholesterol to total lipids ratio in very large HDL	N/A	Ratios	N/A
Cholesteryl esters to total lipids ratio in very large HDL	N/A	Ratios	N/A
Free cholesterol to total lipids ratio in very large HDL	N/A	Ratios	N/A
Triglycerides to total lipids ratio in very large HDL	N/A	Ratios	N/A
Phospholipids to total lipids ratio in large HDL	N/A	Ratios	N/A
Cholesterol to total lipids ratio in large HDL	N/A	Ratios	N/A
Cholesteryl esters to total lipids ratio in large HDL	N/A	Ratios	N/A
Free cholesterol to total lipids ratio in large HDL	N/A	Ratios	N/A
Triglycerides to total lipids ratio in large HDL	N/A	Ratios	N/A
Phospholipids to total lipids ratio in medium HDL	N/A	Ratios	N/A
Cholesterol to total lipids ratio in medium HDL	N/A	Ratios	N/A
Cholesteryl esters to total lipids ratio in medium HDL	N/A	Ratios	N/A
Free cholesterol to total lipids ratio in medium HDL	N/A	Ratios	N/A
Triglycerides to total lipids ratio in medium HDL	N/A	Ratios	N/A
Phospholipids to total lipids ratio in small HDL	N/A	Ratios	N/A
Cholesterol to total lipids ratio in small HDL	N/A	Ratios	N/A
Cholesteryl esters to total lipids ratio in small HDL	N/A	Ratios	N/A
Free cholesterol to total lipids ratio in small HDL	N/A	Ratios	N/A
Triglycerides to total lipids ratio in small HDL	N/A	Ratios	N/A

© 2023 Yang S et al. *JAMA Network Open.*

eTable 2

Metabolic markers used in models discriminating common diseases and mortality.

**T2D**
Ratio of saturated fatty acids to total fatty acids
Ratio of linoleic acid to total fatty acids
Total Lipids in Medium HDL
Phospholipids to total lipids ratio in small HDL
Total Lipids in HDL
Free Cholesterol in HDL
Concentration of HDL Particles
Ratio of omega-6 fatty acids to total fatty acids
Ratio of apolipoprotein B to apolipoprotein A1
HDL Cholesterol
Phospholipids in Large HDL
Cholesteryl Esters in HDL
Total Concentration of Lipoprotein Particles
Ratio of polyunsaturated fatty acids to total fatty acids
Total Lipids in Small HDL
Total Lipids in Large HDL
Phospholipids to total lipids ratio in medium LDL
Phosphatidylcholines
Glucose
Free Cholesterol in Small HDL
Saturated Fatty Acids
Cholesterol to total lipids ratio in small HDL
Total Cholines
Cholesterol to total lipids ratio in small VLDL
Triglycerides to total lipids ratio in small VLDL
**OSAHS**
Ratio of saturated fatty acids to total fatty acids
Ratio of linoleic acid to total fatty acids
Concentration of Medium HDL Particles
Phospholipids to total lipids ratio in small HDL
Cholesterol in Medium HDL
Ratio of omega-6 fatty acids to total fatty acids
Ratio of apolipoprotein B to apolipoprotein A1
HDL Cholesterol
Ratio of polyunsaturated fatty acids to total fatty acids
Phosphoglycerides
Free Cholesterol in Small HDL
Concentration of Large HDL Particles
Saturated Fatty Acids

© 2023 Yang S et al. *JAMA Network Open.*

Total Cholines
**MI**
Ratio of saturated fatty acids to total fatty acids
Phospholipids in Medium HDL
Ratio of linoleic acid to total fatty acids
Phospholipids in HDL
Free Cholesterol in Medium HDL
Apolipoprotein A1
Cholesteryl Esters in Medium HDL
Phospholipids in Small HDL
Ratio of omega-6 fatty acids to total fatty acids
Cholesteryl Esters in HDL
Ratio of polyunsaturated fatty acids to total fatty acids
Total Lipids in Small HDL
Phosphatidylcholines
Glucose
Cholesteryl esters to total lipids ratio in small VLDL
Saturated Fatty Acids
Total Cholines
Triglycerides to total lipids ratio in small VLDL
**HF**
Ratio of linoleic acid to total fatty acids
Free Cholesterol in Medium HDL
Apolipoprotein A1
Cholesterol in Medium HDL
Free Cholesterol in HDL
Phospholipids in Small HDL
Ratio of omega-6 fatty acids to total fatty acids
Phospholipids in Large HDL
Ratio of polyunsaturated fatty acids to total fatty acids
Phosphoglycerides
Total Lipids in Large HDL
Phosphatidylcholines
Glucose
Free Cholesterol in Large HDL
Concentration of Large HDL Particles
Total Cholines
Cholesterol to total lipids ratio in small VLDL
Triglycerides to total lipids ratio in small VLDL
**Dementia**
Phospholipids in Medium HDL
Ratio of linoleic acid to total fatty acids
Total Lipids in Medium HDL

© 2023 Yang S et al. *JAMA Network Open.*

Phospholipids to total lipids ratio in small HDL
Free Cholesterol in Medium HDL
Concentration of HDL Particles
Ratio of apolipoprotein B to apolipoprotein A1
HDL Cholesterol
Phospholipids in Large HDL
Glucose
Cholesteryl esters to total lipids ratio in small VLDL
Free Cholesterol in Small HDL
Cholesterol to total lipids ratio in small HDL
Triglycerides to total lipids ratio in small VLDL
**Stroke**
Phospholipids in Medium HDL
Total Lipids in Medium HDL
Phospholipids to total lipids ratio in small HDL
Free Cholesterol in Medium HDL
Total Lipids in HDL
Phospholipids in Large HDL
Ratio of polyunsaturated fatty acids to total fatty acids
Phosphoglycerides
Glucose
Cholesteryl esters to total lipids ratio in small VLDL
Concentration of Large HDL Particles
Total Cholines
Cholesterol to total lipids ratio in small VLDL
Ratio of saturated fatty acids to total fatty acids
**All-cause mortality**
Ratio of saturated fatty acids to total fatty acids
Phospholipids in Medium HDL
Ratio of linoleic acid to total fatty acids
Total Lipids in Medium HDL
Phospholipids to total lipids ratio in small HDL
Free Cholesterol in Medium HDL
Apolipoprotein A1
Total Lipids in HDL
Cholesterol in Medium HDL
Cholesteryl Esters in Medium HDL
Free Cholesterol in HDL
Ratio of omega-6 fatty acids to total fatty acids
Phospholipids in Large HDL
Ratio of polyunsaturated fatty acids to total fatty acids
Total Lipids in Small HDL
Total Lipids in Large HDL

© 2023 Yang S et al. *JAMA Network Open.*

Phosphatidylcholines
Glucose
Cholesteryl esters to total lipids ratio in small VLDL
Free Cholesterol in Small HDL
Free Cholesterol in Large HDL
Concentration of Large HDL Particles
Saturated Fatty Acids
Cholesterol to total lipids ratio in small HDL
Cholesterol to total lipids ratio in small VLDL
**CVD mortality**
Ratio of saturated fatty acids to total fatty acids
Phospholipids in Medium HDL
Phospholipids to total lipids ratio in small HDL
Cholesteryl Esters in Medium HDL
Phospholipids in Large HDL
Ratio of polyunsaturated fatty acids to total fatty acids
Phosphoglycerides
Phosphatidylcholines
Glucose
Cholesteryl esters to total lipids ratio in small VLDL
Free Cholesterol in Small HDL
Free Cholesterol in Large HDL
Triglycerides to total lipids ratio in small VLDL
**Cancer mortality**
Ratio of saturated fatty acids to total fatty acids
Phospholipids in Medium HDL
Phospholipids to total lipids ratio in small HDL
Free Cholesterol in Medium HDL
Apolipoprotein A1
Phospholipids in Large HDL
Ratio of polyunsaturated fatty acids to total fatty acids
Total Lipids in Small HDL
Phosphatidylcholines
Glucose
Cholesteryl esters to total lipids ratio in small VLDL
Free Cholesterol in Small HDL
Free Cholesterol in Large HDL
Cholesterol to total lipids ratio in small VLDL
**Other mortality**
Ratio of saturated fatty acids to total fatty acids
Phospholipids in Medium HDL
Phospholipids to total lipids ratio in small HDL
Free Cholesterol in Medium HDL

© 2023 Yang S et al. *JAMA Network Open.*

Apolipoprotein A1
Total Lipids in HDL
Phospholipids in Small HDL
Ratio of apolipoprotein B to apolipoprotein A1
Cholesteryl Esters in HDL
Ratio of polyunsaturated fatty acids to total fatty acids
Phospholipids to total lipids ratio in medium LDL
Phosphatidylcholines
Cholesteryl esters to total lipids ratio in small VLDL
Free Cholesterol in Large HDL
Concentration of Large HDL Particles
Saturated Fatty Acids
Cholesterol to total lipids ratio in small HDL
Cholesterol to total lipids ratio in small VLDL

© 2023 Yang S et al. *JAMA Network Open.*

eTable 3

Baseline characteristics of the study population from the UKB cohort.

Characteristic	Total	Population-I	Population-II		P value § †	P value § ‡
			Discovery set	Validation set		
Number of subjects	93838	7824	60095	25919	-	-
Age at recruitment						
≤49	21438 (22.8)	2248 (28.7)	13404 (22.3)	5786 (22.3)	**<0.001**	0.763
50-54	13843 (14.8)	1191 (15.2)	8779 (14.6)	3873 (14.9)		
55-59	16856 (18.0)	1319 (16.9)	10863 (18.1)	4674 (18.0)		
60-64	23246 (24.8)	1800 (23.0)	15003 (25.0)	6443 (24.9)		
≥65	18455 (19.7)	1266 (16.2)	12046 (20.0)	5143 (19.8)		
Gender						
Female	51182 (54.5)	4122 (52.7)	32931 (54.8)	14129 (54.5)	**0.002**	0.444
Male	42656 (45.5)	3702 (47.3)	27164 (45.2)	11790 (45.5)		
Race						
White	88754 (94.6)	7188 (91.9)	56993 (94.8)	24573 (94.8)	**<0.001**	0.857
Others	4652 (5.0)	589 (7.5)	2838 (4.7)	1225 (4.7)		
Missing	432 (0.5)	47 (0.6)	264 (0.4)	121 (0.5)		
Townsend deprivation index						
Quantile 1	23598 (25.1)	1749 (22.4)	15269 (25.4)	6580 (25.4)	**<0.001**	0.662
Quantile 2	23394 (24.9)	1861 (23.8)	14969 (24.9)	6564 (25.3)		
Quantile 3	23324 (24.9)	2145 (27.4)	14866 (24.7)	6313 (24.4)		
Quantile 4	23397 (24.9)	2059 (26.3)	14912 (24.8)	6426 (24.8)		
Missing	125 (0.1)	10 (0.1)	79 (0.1)	36 (0.1)		

© 2023 Yang S et al. *JAMA Network Open.*

Average total household income before tax (£)						
< 18k	18820 (20.1)	1214 (15.5)	12308 (20.5)	5298 (20.4)	**<0.001**	0.782
18k~30k	20770 (22.1)	1627 (20.8)	13442 (22.4)	5701 (22.0)		
31k~51k	20594 (21.9)	1846 (23.6)	13065 (21.7)	5683 (21.9)		
52k~100k	15728 (16.8)	1610 (20.6)	9811 (16.3)	4307 (16.6)		
> 100k	3982 (4.2)	516 (6.6)	2430 (4.0)	1036 (4.0)		
Missing	13944 (14.9)	1011 (12.9)	9039 (15.0)	3894 (15.0)		
Education achievement						
Level O	32411 (34.5)	2316 (29.6)	21019 (35.0)	9076 (35.0)	**<0.001**	0.798
Level A	4934 (5.3)	499 (6.4)	3124 (5.2)	1311 (5.1)		
University	55406 (59.0)	5009 (64.0)	35185 (58.5)	15212 (58.7)		
Missing	1087 (1.2)	0 (0.0)	767 (1.3)	320 (1.2)		
Body mass index						
Normal	30120 (32.1)	2650 (33.9)	19239 (32.0)	8231 (31.8)	**0.001**	0.331
Overweight	39832 (42.4)	3311 (42.3)	25401 (42.3)	11120 (42.9)		
Obesity	23514 (25.1)	1839 (23.5)	15206 (25.3)	6469 (25.0)		
Missing	372 (0.4)	24 (0.3)	249 (0.4)	99 (0.4)		
Smoking						
Never	32963 (35.1)	2711 (34.6)	21198 (35.3)	9054 (34.9)	**0.018**	0.454
Ever/Current	10110 (10.8)	771 (9.9)	6547 (10.9)	2792 (10.8)		
Missing	50765 (54.1)	4342 (55.5)	32350 (53.8)	14073 (54.3)		
Drinking						
Never	3510 (3.7)	261 (3.3)	2294 (3.8)	955 (3.7)	**0.040**	0.387
Ever/Current	85974 (91.6)	7235 (92.5)	55018 (91.6)	23721 (91.5)		
Missing	4354 (4.6)	328 (4.2)	2783 (4.6)	1243 (4.8)		

© 2023 Yang S et al. *JAMA Network Open.*

Spherical equivalent, diopter	-0.05 ± 1.88	-0.05 ± 1.88	-	-	-	-
Intraocular pressure, mmHg	15.21 ± 2.90	15.21 ± 2.90	-	-	-	-
Lipid-lowering medication						
No	76961 (82.0)	6525 (83.4)	49185 (81.8)	21251 (82.0)	**0.003**	0.620
Yes	16877 (18.0)	1299 (16.6)	10910 (18.2)	4668 (18.0)		
Antihypertensive medication						
No	73674 (78.5)	6424 (82.1)	47038 (78.3)	20212 (78.0)	**<0.001**	0.347
Yes	20164 (21.5)	1400 (17.9)	13057 (21.7)	5707 (22.0)		
Insulin						
No	93056 (99.2)	7772 (99.3)	59585 (99.2)	25699 (99.2)	0.230	1.000
Yes	782 (0.8)	52 (0.7)	510 (0.8)	220 (0.8)		

§ Bold indicates statistically significant.

† Comparison of characteristics between population-I and population-II.

‡ Comparison of characteristics between discovery set and validation set in population-II.

© 2023 Yang S et al. *JAMA Network Open.*

eTable 4

Baseline characteristics of the study population from the GDES cohort.

**Characteristic**	**Total**	**Non-CVD**	**Incident-CVD**	**P value §**
Number of subjects	592	493	99	
Age at recruitment				
≤49	37 (6.2)	35 (7.1)	2 (2.0)	0.174
50-54	33 (5.6)	28 (5.7)	5 (5.1)	
55-59	92 (15.5)	81 (16.4)	11 (11.1)	
60-64	146 (24.7)	119 (24.1)	27 (27.3)	
≥65	284 (48.0)	230 (46.7)	54 (54.5)	
Gender				
Female	345 (58.3)	287 (58.2)	58 (58.6)	1
Male	247 (41.7)	206 (41.8)	41 (41.4)	
Education				
Elementary school or below	41 (6.9)	33 (6.7)	8 (8.1)	0.723
Middle school	298 (50.3)	249 (50.5)	49 (49.5)	
University	126 (21.3)	102 (20.7)	24 (24.2)	
Missing	127 (21.5)	109 (22.1)	18 (18.2)	
Body mass index				
Normal	256 (43.2)	216 (43.8)	40 (40.4)	0.861
Overweight	256 (43.2)	212 (43.0)	44 (44.4)	
Obesity	79 (13.3)	64 (13.0)	15 (15.2)	
Missing	1 (0.2)	1 (0.2)	0 (0.0)	
Smoking				
Never	465 (78.5)	382 (77.5)	83 (83.8)	0.204
Ever/Current	127 (21.5)	111 (22.5)	16 (16.2)	
Drinking				
Never	478 (80.7)	397 (80.5)	81 (81.8)	0.875
Ever/Current	114 (19.3)	96 (19.5)	18 (18.2)	
Hypertension				
No	367 (62.0)	353 (71.6)	14 (14.1)	**<0.001**
Yes	225 (38.0)	140 (28.4)	85 (85.9)	
Insulin				
No	467 (78.9)	392 (79.5)	75 (75.8)	0.484
Yes	125 (21.1)	101 (20.5)	24 (24.2)	

§ Bold indicates statistically significant.

© 2023 Yang S et al. *JAMA Network Open.*

eTable 5

Significant metabolites associated with GCIPLT.

Metabolites	β	95%CI		P §	P_{BH} †
Ratio of saturated fatty acids to total fatty acids	-0.403	-0.568	-0.238	1.73×10^{-6}	2.17×10^{-4}
Phospholipids in Medium HDL	-0.288	-0.414	-0.161	8.48×10^{-6}	5.31×10^{-4}
Ratio of linoleic acid to total fatty acids	0.324	0.174	0.475	2.43×10^{-5}	7.09×10^{-4}
Total Lipids in Medium HDL	-0.275	-0.403	-0.147	2.57×10^{-5}	7.16×10^{-4}
Phospholipids in HDL	-0.281	-0.413	-0.149	2.92×10^{-5}	7.31×10^{-4}
Concentration of Medium HDL Particles	-0.269	-0.398	-0.141	4.04×10^{-5}	7.91×10^{-4}
Phospholipids to total lipids ratio in small HDL	-0.256	-0.379	-0.133	4.52×10^{-5}	8.09×10^{-4}
Free Cholesterol in Medium HDL	-0.267	-0.397	-0.136	6.04×10^{-5}	9.45×10^{-4}
Apolipoprotein A1	-0.256	-0.386	-0.126	1.15×10^{-4}	1.39×10^{-3}
Total Lipids in HDL	-0.261	-0.395	-0.128	1.28×10^{-4}	1.47×10^{-3}
Cholesterol in Medium HDL	-0.254	-0.385	-0.124	1.31×10^{-4}	1.49×10^{-3}
Cholesteryl Esters in Medium HDL	-0.251	-0.381	-0.121	1.57×10^{-4}	1.64×10^{-3}
Free Cholesterol in HDL	-0.240	-0.375	-0.105	5.02×10^{-4}	4.31×10^{-3}
Phospholipids in Small HDL	-0.209	-0.328	-0.089	6.46×10^{-4}	5.16×10^{-3}
Concentration of HDL Particles	-0.218	-0.343	-0.092	6.69×10^{-4}	5.29×10^{-3}
Ratio of omega-6 fatty acids to total fatty acids	0.258	0.109	0.408	7.20×10^{-4}	5.56×10^{-3}
Ratio of apolipoprotein B to apolipoprotein A1	0.221	0.091	0.350	8.23×10^{-4}	6.06×10^{-3}
HDL Cholesterol	-0.224	-0.361	-0.087	1.31×10^{-3}	8.88×10^{-3}
Phospholipids in Large HDL	-0.224	-0.362	-0.086	1.46×10^{-3}	9.64×10^{-3}
Cholesteryl Esters in HDL	-0.218	-0.354	-0.081	1.78×10^{-3}	1.11×10^{-2}
Total Concentration of Lipoprotein Particles	-0.196	-0.321	-0.070	2.27×10^{-3}	1.36×10^{-2}
Ratio of polyunsaturated fatty acids to total fatty acids	0.229	0.077	0.382	3.25×10^{-3}	1.78×10^{-2}
Total Lipids in Small HDL	-0.172	-0.290	-0.053	4.65×10^{-3}	2.28×10^{-2}
Phosphoglycerides	-0.182	-0.309	-0.056	4.69×10^{-3}	2.30×10^{-2}
Total Lipids in Large HDL	-0.202	-0.342	-0.061	4.84×10^{-3}	2.34×10^{-2}
Phospholipids to total lipids ratio in medium LDL	0.170	0.048	0.292	6.23×10^{-3}	2.74×10^{-2}
Phosphatidylcholines	-0.178	-0.306	-0.050	6.42×10^{-3}	2.78×10^{-2}
Glucose	-0.183	-0.315	-0.051	6.52×10^{-3}	2.81×10^{-2}
Cholesteryl esters to total lipids ratio in small VLDL	0.168	0.046	0.291	7.06×10^{-3}	2.94×10^{-2}

© 2023 Yang S et al. *JAMA Network Open.*

Free Cholesterol in Small HDL	-0.164	-0.284	-0.044	7.29×10^{-3}	2.99×10^{-2}
Free Cholesterol in Large HDL	-0.187	-0.326	-0.049	7.88×10^{-3}	3.12×10^{-2}
Concentration of Large HDL Particles	-0.187	-0.325	-0.049	8.11×10^{-3}	3.17×10^{-2}
Saturated Fatty Acids	-0.170	-0.297	-0.043	8.60×10^{-3}	3.26×10^{-2}
Cholesterol to total lipids ratio in small HDL	0.158	0.035	0.281	1.21×10^{-2}	4.22×10^{-2}
Total Cholines	-0.162	-0.290	-0.034	1.32×10^{-2}	4.47×10^{-2}
Cholesterol to total lipids ratio in small VLDL	0.161	0.033	0.289	1.38×10^{-2}	4.62×10^{-2}
Triglycerides to total lipids ratio in small VLDL	-0.160	-0.287	-0.032	1.43×10^{-2}	4.74×10^{-2}

§Adjusted for sex, age, race, education, Townsend deprivation index, household income, body mass index, smoking, alcohol, lipid-lowering medication, spherical equivalent, and intraocular pressure.

†Benjamini-Hochberg corrected P value.

GCIPLT = ganglion cell-inner plexiform layer thickness; HDL = high-density lipoprotein; LDL = low-density lipoprotein; VLDL = very low-density lipoprotein; CI = confidence interval.

© 2023 Yang S et al. *JAMA Network Open.*

eTable 6

No. of incident health outcomes in total, discovery set, and validation set.

Outcome	Total	Discovery set	Validation set
**Common diseases**			
T2D	6,071 (7.06%)	4,215 (7.01%)	1,856 (7.16%)
MI	2,866 (3.33%)	1,988 (3.31%)	878 (3.39%)
HF	2,537 (2.95%)	1,756 (2.92%)	781 (3.01%)
Stroke	1,578 (1.83%)	1,073 (1.79%)	505 (1.95%)
Dementia	1,219 (1.42%)	828 (1.38%)	391 (1.51%)
OSAHS	1,366 (1.59%)	923 (1.54%)	443 (1.71%)
**Mortality**			
All-cause mortality	6,254 (7.27%)	4,370 (7.27%)	1,884 (7.27%)
CVD mortality	1,544 (1.80%)	1,068 (1.78%)	476 (1.84%)
Cancer mortality	3,151 (3.66%)	2,202 (3.66%)	949 (3.66%)
Other mortality	1,559 (1.81%)	1,100 (1.83%)	459 (1.77%)

T2D = type 2 diabetes; MI = myocardial infarction; HF = heart failure; OSAHS = obstructive sleep apnea/hypopnea syndrome; CVD = cardiovascular disease.

© 2023 Yang S et al. *JAMA Network Open.*

eTable 7

Discriminative power of clinical indicators and GCIPLT metabolic profiles for predicting mortality and common diseases.

Endpoints	C-statistic (95%CI)			P value§
	Model 1*	Model 2†	Model 3‡	
**Morbidity**				
T2D	0.803 (0.792-0.814)	0.824 (0.812-0.836)	0.862 (0.852-0.872)	$<1.0\times10^{-8}$
MI	0.768 (0.751-0.786)	0.739 (0.719-0.758)	0.792 (0.775-0.808)	$<1.0\times10^{-8}$
HF	0.790 (0.773-0.807)	0.700 (0.678-0.722)	0.803 (0.786-0.820)	9.60×10^{-5}
Stroke	0.719 (0.693-0.745)	0.656 (0.629-0.683)	0.739 (0.714-0.764)	1.58×10^{-4}
OSAHS	0.748 (0.719-0.777)	0.720 (0.694-0.746)	0.758 (0.734-0.783)	0.015
Dementia	0.804 (0.780-0.828)	0.652 (0.619-0.684)	0.811 (0.786-0.834)	0.128
**Mortality**				
All-cause	0.724 (0.711-0.738)	0.668 (0.653-0.682)	0.747 (0.734-0.760)	$<1.0\times10^{-8}$
CVD	0.763 (0.739-0.788)	0.720 (0.693-0.747)	0.790 (0.767-0.812)	2.51×10^{-5}
Cancer	0.682 (0.664-0.701)	0.629 (0.609-0.650)	0.701 (0.683-0.720)	9.42×10^{-6}
Other	0.750 (0.725-0.774)	0.681 (0.651-0.710)	0.776 (0.752-0.800)	8.47×10^{-4}

*Clinical indicators-based model for predicting T2D includes age, sex, Townsend deprivation index, smoking, drinking, ethnicity, BMI, WHR, blood pressure-lowering medication, and family history of diabetes. Clinical indicators-based model for predicting MI includes age, sex, ethnicity, smoking, drinking, BMI, hypertension, diabetes, total cholesterol, and LDL-c. Clinical indicators-based model for predicting HF includes age, sex, ethnicity, smoking, drinking, BMI, ACR, HbA1c, hemoglobin, cardiovascular diseases, and family history of CVD. Clinical indicators-based model for predicting stroke includes age, sex, ethnicity, smoking, drinking, BMI, hypertension, family history of stroke, total cholesterol, and SBP. Clinical indicators-based model for predicting mortality includes age, sex, Townsend deprivation index, smoking, drinking,

© 2023 Yang S et al. *JAMA Network Open.*

BMI, physical activity, lipid-lowering medication, SBP, diabetes, and total cholesterol. Clinical indicators-based model for predicting dementia includes age, sex, ethnicity, smoking, drinking, education, BMI, stroke, family history of dementia, and apoE4 allele. Clinical indicators-based model for predicting OSAHS includes age, sex, smoking, drinking, ethnicity, BMI, WHR, hypertension, and diabetes.

†GCIPLT metabolic state models based on GCIPLT-related metabolites.

‡Combined models based on clinical indicators and GCIPLT-related metabolites.

§Compared between model 3 and model 1.

T2D = type 2 diabetes; MI = myocardial infarction; HF = heart failure; OSAHS = obstructive sleep apnea/hypopnea syndrome; CVD = cardiovascular disease; BMI = body mass index; WHR = waist-to-hip ratio; ACR = microalbumin/creatinine ratio; HbA1c = glycosylated hemoglobin A1c; SBP = systolic blood pressure; apoE4 = apolipoprotein E4.

© 2023 Yang S et al. *JAMA Network Open.*

eTable 8

NRIs improvements of incorporating GCIPLT metabolic profiles for mortality and morbidity of common diseases.

Outcome	NRI (Point estimation)	NRI (95% CI) †		P
**Common diseases**				
T2D	0.283	0.243	0.315	<0.001
MI	0.085	0.050	0.128	<0.001
HF	0.053	0.023	0.101	<0.01
Stroke	0.084	0.027	0.156	<0.01
Dementia	0.073	0.002	0.127	<0.01
OSAHS	0.058	0.014	0.129	0.016
**Mortality**				
All-cause mortality	0.101	0.073	0.140	<0.001
CVD mortality	0.110	0.071	0.185	<0.001
Cancer mortality	0.067	0.048	0.137	<0.001
Other mortality	0.120	0.072	0.188	<0.001

NRI = net reclassification index; GCIPLT = ganglion cell-inner plexiform layer thickness; T2D = type 2 diabetes; MI = myocardial infarction; HF = heart failure; OSAHS = obstructive sleep apnea/hypopnea syndrome; CVD = cardiovascular disease; CI = confidential interval.

† CIs were estimated using bootstrap.

© 2023 Yang S et al. *JAMA Network Open.*

eTable 9

List summarizing metabolic profiles identified in the GDES cohort using LC/MS profiling.

Compounds	Group	Subgroup	β	95%CI		P
1-Methylinosine	Nucleotide and Its metabolites	Nucleotide and Its metabolites	-1.120	-1.627	-0.613	1.76E-05
N6-Succinyl Adenosine	Nucleotide and Its metabolites	Nucleotide and Its metabolites	-1.108	-1.610	-0.606	1.80E-05
N-acetylpyrrolidine	Heterocyclic compounds	Heterocyclic compounds	-1.142	-1.677	-0.608	3.25E-05
2-(Dimethylamino)Guanosine	Nucleotide and Its metabolites	Nucleotide and Its metabolites	-1.074	-1.579	-0.569	3.56E-05
N6-(2-Hydroxyethyl)adenosine	Nucleotide and Its metabolites	Nucleotide and Its metabolites	-1.056	-1.561	-0.551	4.79E-05
Acetylvaline	Amino acid and Its metabolites	Amino acid derivatives	-1.070	-1.583	-0.557	4.97E-05
6-O-methylguanine	Nucleotide and Its metabolites	Nucleotide and Its metabolites	-1.063	-1.592	-0.534	9.27E-05
Creatinine	Organic acid and Its derivatives	Organic acid and Its derivatives	-1.080	-1.623	-0.538	1.06E-04
N-Acetyl-L-alanine	Amino acid and Its metabolites	Amino acid derivatives	-1.028	-1.545	-0.512	1.07E-04
N-acetyl-beta-alanine	Amino acid and Its metabolites	Amino acid derivatives	-1.006	-1.521	-0.491	1.42E-04
2-Amino-4,6-pteridinediol	Nucleotide and Its metabolites	Nucleotide and Its metabolites	-0.987	-1.503	-0.472	1.91E-04
(3-Methoxy-4-hydroxyphenyl) ethylene glycol sulfate	Organic acid and Its derivatives	Organic acid and Its derivatives	-0.938	-1.436	-0.440	2.45E-04
B-Pseudouridine	Nucleotide and Its metabolites	Nucleotide and Its metabolites	-0.953	-1.459	-0.446	2.50E-04
Indole-3-acetamide	Heterocyclic compounds	Indole and Its derivatives	-0.934	-1.446	-0.421	3.83E-04
Quinoline-2-carboxylic acid	Heterocyclic compounds	Pteridines and derivatives	-0.910	-1.410	-0.409	3.97E-04
2-Hydroxy-3-Methyl Butanoic Acid	Organic acid and Its derivatives	Organic acid and Its derivatives	-0.900	-1.407	-0.393	5.47E-04
2-Hydroxy-2-Methyl Butyric acid	Organic acid and Its derivatives	Organic acid and Its derivatives	-0.886	-1.394	-0.378	6.73E-04
Quinoline-4-carboxylic acid	Heterocyclic compounds	Pteridines and derivatives	-0.916	-1.441	-0.391	6.75E-04
Kynurenic Acid	Amino acid and Its metabolites	Amino acid derivatives	-0.842	-1.339	-0.345	9.57E-04
3-Hydroxy-3-Methylpentane-1,5-Dioic	Amino acid and Its metabolites	Amino acid derivatives	-0.850	-1.354	-0.346	1.01E-03

© 2023 Yang S et al. *JAMA Network Open.*

Acid						
5-Hydroxy-2'-deoxyuridine	Nucleotide and Its metabolites	Nucleotide and Its metabolites	-0.846	-1.357	-0.336	1.22E-03
Carnitine C6-2OH	FA	CAR	-0.823	-1.327	-0.319	1.45E-03
S-(5-Adenosy)-L-Homocysteine	Amino acid and Its metabolites	Amino acid derivatives	-0.829	-1.338	-0.319	1.51E-03
Hydroxyphenyllactic acid	Organic acid and Its derivatives	Organic acid and Its derivatives	-0.852	-1.376	-0.327	1.53E-03

© 2023 Yang S et al. *JAMA Network Open.*

eTable 10

Sensitivity analysis of excluding all missing values.

Endpoints	C-statistic (95%CI)			P value§
	Model 1*	Model 2†	Model 3‡	
**Morbidity**				
T2D	0.791 (0.775-0.807)	0.811 (0.793-0.828)	0.849 (0.834-0.864)	$<1.0\times10^{-8}$
MI	0.762 (0.739-0.785)	0.733 (0.708-0.758)	0.784 (0.763-0.805)	2.19×10^{-6}
HF	0.784 (0.760-0.807)	0.699 (0.670-0.728)	0.798 (0.775-0.821)	9.60×10^{-5}
Stroke	0.723 (0.691-0.755)	0.639 (0.603-0.675)	0.743 (0.711-0.774)	0.007
OSAHS	0.750 (0.708-0.792)	0.709 (0.672-0.745)	0.759 (0.725-0.794)	0.056
Dementia	0.810 (0.778-0.843)	0.648 (0.605-0.692)	0.817 (0.784-0.850)	0.150
**Mortality**				
All-cause	0.742 (0.725-0.759)	0.681 (0.661-0.700)	0.761 (0.744-0.778)	3.45×10^{-7}
CVD	0.771 (0.742-0.799)	0.716 (0.681-0.751)	0.791 (0.762-0.819)	0.007
Cancer	0.693 (0.667-0.718)	0.643 (0.616-0.670)	0.712 (0.687-0.736)	9.08×10^{-4}
Other	0.768 (0.738-0.799)	0.696 (0.659-0.734)	0.791 (0.762-0.821)	0.015

*Clinical indicators-based model for predicting T2D includes age, sex, Townsend deprivation index, smoking, drinking, ethnicity, BMI, WHR, blood pressure-lowering medication, and family history of diabetes. Clinical indicators-based model for predicting MI includes age, sex, ethnicity, smoking, drinking, BMI, hypertension, diabetes, total cholesterol, and LDL-c. Clinical indicators-based model for predicting HF includes age, sex, ethnicity, smoking, drinking, BMI, ACR, HbA1c, hemoglobin, cardiovascular diseases, and family history of CVD. Clinical indicators-based model for predicting stroke includes age, sex, ethnicity, smoking, drinking, BMI, hypertension, family history of stroke, total cholesterol, and SBP. Clinical indicators-based model for predicting mortality includes age, sex, Townsend deprivation index, smoking, drinking,

© 2023 Yang S et al. *JAMA Network Open.*

BMI, physical activity, lipid-lowering medication, SBP, diabetes, and total cholesterol. Clinical indicators-based model for predicting dementia includes age, sex, ethnicity, smoking, drinking, education, BMI, stroke, family history of dementia, and apoE4 allele. Clinical indicators-based model for predicting OSAHS includes age, sex, smoking, drinking, ethnicity, BMI, WHR, hypertension, and diabetes.

†GCIPLT metabolic state models based on GCIPLT-related metabolites.

‡Combined models based on clinical indicators and GCIPLT-related metabolites.

§Compared between model 3 and model 1.

T2D = type 2 diabetes; MI = myocardial infarction; HF = heart failure; OSAHS = obstructive sleep apnea/hypopnea syndrome; CVD = cardiovascular disease; BMI = body mass index; WHR = waist-to-hip ratio; ACR = microalbumin/creatinine ratio; HbA1c = glycosylated hemoglobin A1c; SBP = systolic blood pressure; apoE4 = apolipoprotein E4.

© 2023 Yang S et al. *JAMA Network Open.*

eMethods

Eligibility criteria

A total of 93,838 participants from the UKB cohort were included for final analysis in this study, which comprised 7,824 for phase-I analysis and 86,014 for phase-II analysis. Phase-I analysis included participants who underwent both eligible retinal OCT measurements and metabolomic profiling at baseline assessment. Details were as follows. In accordance with established quality control standards, we excluded eyes with missing thickness values (1,350 right eyes and 1,791 left eyes), low signal strength (Q<45) (6,105 right eyes and 5,149 left eyes), poor centration or segmentation (poorest 20% indicators) (19,780 right eyes and 20,022 left eyes) from a total of 67,135 participants underwent OCT scanning at baseline. To minimize the impact of other ocular parameters, we also excluded eyes with high refractive error (spherical equivalent [SE] >6 or <-6 diopters [D]) (1,633 right eyes and 1,833 left eyes), visual impairment (> 0.1 logarithm of the minimum angle of resolution [logMAR]) (9,345 right eyes and 8,863 left eyes), or abnormal intraocular pressure (IOP) (≥ 22 or ≤ 5 mmHg) (3,148 right eyes and 3,551 left eyes). Additionally, we excluded patients with glaucoma (400 participants), other retinal disorders (703 participants), and neurodegenerative diseases (111 participants) due to the potential secondary RNFL destruction. Finally, participants without metabolomics profiling were further excluded. This resulted in a total of 7,824 participants for phase-I analysis. The medical history of participants was obtained through questionnaires, interviews, and inpatient diagnoses prior to baseline. Participants who did not complete metabolomic profiling were further excluded.

Phase-II analysis included 110,730 participants who complete metabolomic profiling at baseline assessment, with the exclusion of participants who lack hospital records (18,032 participants). Meanwhile, those who already included in phase-I analysis (6,684 participants) were further excluded. Thus, the participants for phase-II analysis and those for phase-I analysis are two separate subsets that do not overlap with each other. This resulted in a total of 86,014 participants for phase-II analysis.

© 2023 Yang S et al. *JAMA Network Open.*

Participants with previous diagnosis were further excluded for each analysis (e.g., in the case of T2D endpoint analysis, participants diagnosed with T2D at baseline were excluded).

Profiling details and quality control processes of 1H-NMR profiling

Details about the metabolomic profiling protocol have been described elsewhere.[1] Sample collection was undertaken at baseline in 22 local assessment centers across the UK between 2007 and 2010. The blood sample handling and storage protocol has been previously described.[2] The metabolomic profiling took place in Finland between 2019 and 2020 using six NMR spectrometers. Accredited quality control was done during the whole process to eliminate systemic and technical variance, and only samples and biomarkers that underwent the quality control process were stored in the UK Biobank dataset and used in our present study.

EDTA plasma samples from aliquot 3 were prepared in 96-well plates by UK Biobank laboratory (Stockport, UK). TECAN freedom EVO 150 robotic liquid handlers were used for aliquoting plasma samples. The plasma samples were shipped in batches to Nightingale Health laboratories in Finland on dry ice. Before preparation, frozen samples were thawed slowly, mixed gently and centrifuged. Aliquots of each sample were mixed with a phosphate buffer and subjected to measurement using six 500 MHz NMR spectrometers. Nightingale Health's proprietary software (quantification library 2020) was used to quantify the biomarkers.

Each 96-well plate included two internal control samples provided by Nightingale Health to track consistency across multiple spectrometers. Four sets of internal control samples with different biomarker concentration span were used across 1,352 96-well plates and interleaved between NMR instruments for extended periods. Blind duplicate samples from UK Biobank were included on each 96-well plate, with position revealed only after results delivery. The coefficient of variation

© 2023 Yang S et al. *JAMA Network Open.*

(CV) distributions for the blinded replicates and Nightingale Health's internal control samples were typically below 5%. These results met pre-specified CV targets for each set of approximately 20,000 consecutively measured samples.

The NMR biomarker data in the UK Biobank can generally be used for epidemiological analyses without any preprocessing and can in principle be analyzed in the same manner as the clinical chemistry data available in UKB. Biomarker values substantially affected by interfering substances have been removed during the quality control procedures.

Reference

1. Julkunen, H., Cichońska, A., Tiainen, M., Koskela, H., Nybo, K., Mäkelä, V., Nokso-Koivisto, J., Kristiansson, K., Perola, M., Salomaa, V., Jousilahti, P., Lundqvist, A., Kangas, A. J., Soininen, P., Barrett, J. C., & Würtz, P. (2023). Atlas of plasma NMR biomarkers for health and disease in 118,461 individuals from the UK Biobank. Nature communications, 14(1), 604.
2. Elliott, P., Peakman, T. C., & UK Biobank (2008). The UK Biobank sample handling and storage protocol for the collection, processing and archiving of human blood and urine. International journal of epidemiology, 37(2), 234–244.

Quality control of SD-OCT imaging in the UKB cohort

Quality control measures for scanning included the image quality score, ILM indicator, validity count, and motion indicators. The IQS measures the signal strength, while the ILM indicator identifies blinks and segmentation errors. The validity count identifies significant clipping in the OCT scan's z-axis dimension. The motion indicators use Pearson correlations and absolute differences of the nerve fiber layer and full retinal thicknesses from each set of consecutive B-scans to identify blinks, eye-motion artifacts, and segmentation failures.

© 2023 Yang S et al. *JAMA Network Open.*

Assessments of covariates in the UKB cohort

At baseline (2006–2010), physical measurements, face-to-face interviews, and detailed self-administered touchscreen questionnaires were conducted on all participants. The questionnaires included demographic and socioeconomic factors (age, sex, race, education level, Townsend deprivation index, and income), lifestyle factors (smoking and drinking status, and physical activity time per week), family history, and medical history, including the use of insulin, lipid-lowering medications, antihypertensive drugs, and disease diagnosis. Baseline diseases were defined using questionnaires, interviews, and inpatient data based on the ICD-10 codes. Physical measurements including baseline body mass index (BMI), waist-to-hip ratio, blood pressure, visual acuity, refractive error, spherical equivalent, intraocular pressure (IOP), total cholesterol, low-density lipoprotein cholesterol, high-density lipoprotein cholesterol levels, glycosylated hemoglobin A1c, urine microalbumin, and creatinine were obtained. The presence of apolipoprotein E (apoE) ε4 allele was defined using the apoE ε4+ dominant model of ε3/ε4 and ε4/ε4. Detailed information is as followed.

In brief, age at baseline assessment were divided into five categories, including < 50 years, 50~54 years, 55~59 years, 60~64 years, and > 64 years. Townsend deprivation index (TDI) were assigned according to participants' postal codes, which reflected the proportions of unemployment, crowding household, non-car ownership, and homelessness in corresponding output areas. Four quantiles were categorized in the ascending order for TDI (< -3.6, 3.6~-2.1, -2.1~0.6, > 0.6, and missing). Body mass index (BMI) were constructed from height and weight, which were measured by Seca 240cm height measure and Tanita BC418MA body composition analyzer at baseline respectively, and was divided into four categories, including < 25, 25~29.9, ≥30.0, and missing. In the touchscreen questionnaire, UK Biobank participants were asked about their ethnic group, including White, Mixed, Asian, Black, Chinese, and missing. Due to the small number of participants, the last five alternatives were

© 2023 Yang S et al. *JAMA Network Open.*

assembled into others. The average total household income before tax were directly derived from questionnaires, including < £18,000, £18,000 to £30,999, £31,000 to £51,999, £52,000 to £100,000, > £100,000, and missing. Educational qualifications reflected the highest diploma achieved which were divided into three categories including O levels or equivalent, A levels or equivalent, college or university degree, and missing. Time spent on moderate to vigorous activity (MVPA) was categorized into four quantiles based on adapted questions from the short International Physical Activity Questionnaire. Weighted by expended energy, MVPA time were transformed into metabolic Equivalent Task (MET) minutes/week and categorized into four quantiles and missing. For smoking status, previous smokers and current smokers were distinguished from those who never smoked tobacco. Similarly, previous and current drinkers were also separated from those who never drank alcohol. Sensitivity analyses were also conducted by excluding all missing values. The study results can be found in *Supplementary eTable S9*.

Blood biochemistry assessments: Analysis of serum biomarkers utilized 10 immunoassay analyzers (6x DiaSorin Liaison XL & 4x Beckman Coulter DXI 800 and 4 clinical chemistry analyzers (2x Beckman Coulter AU5800 & 2x Siemens Advia 1800).

HbA1c assessment: The HbA1c assay was performed using five Bio-Rad Variant II Turbo analyzers. The five analyzers underwent a rigorous validation protocol including matrix validation for the use of PRBC an atypical matrix for this CE marked assay, to ensure they were all compliant with ISO 17025:2005 standards before analysis commenced. The validation study also included a multi-instrument comparison to ensure five instruments were all in agreement. The Bio-Rad Variant II Turbo Hemoglobin Testing System uses HPLC to determine the relative concentration of HbA1c in PRBCs.

Urine assays: All tests were carried out on a single Beckman Coulter AU5400 clinical chemistry analyzer using the manufacturer's reagents and calibrators except urinary

© 2023 Yang S et al. *JAMA Network Open.*

microalbumin which used reagents and calibrators sourced from Randox Bioscience, UK. The Beckman Coulter AU5400 series clinical chemistry analyzer uses a photometric measurement for the determination of creatinine and microalbumin concentration and a potentiometric measurement for the determination of sodium and potassium concentration.

apoE ε4 allele assessment: Genotyping was conducted by Affymetrix using a bespoke BiLEVE Axiom array or the UK Biobank Axiom array.[1] All genetic data were quality controlled and imputed by UK Biobank. apoE genotype was directly genotyped based on two SNPs (rs7412 and rs429358). apoE ε4+ dominant model of ε3/e4 and ε4/ε4 was used to define the presence of apoE ε4.[2]

Reference

1. Bycroft, C., Freeman, C., Petkova, D., Band, G., Elliott, L. T., Sharp, K., Motyer, A., Vukcevic, D., Delaneau, O., O'Connell, J., Cortes, A., Welsh, S., Young, A., Effingham, M., McVean, G., Leslie, S., Allen, N., Donnelly, P., & Marchini, J. (2018). The UK Biobank resource with deep phenotyping and genomic data. Nature, 562(7726), 203–209.
2. Shang, X., Zhu, Z., Zhang, X., Huang, Y., Zhang, X., Liu, J., Wang, W., Tang, S., Yu, H., Ge, Z., Yang, X., & He, M. (2022). Association of a wide range of chronic diseases and apolipoprotein E4 genotype with subsequent risk of dementia in community-dwelling adults: A retrospective cohort study. EClinicalMedicine, 45, 101335.

SS-OCT imaging protocols and quality control processes in the GDES cohort

After dilatation, structural images of the macula were obtained using a commercially available SS-OCT device (DRI OCT Triton; Topcon, Japan). The instrument has an axial and lateral resolution of 8 and 20 μm, respectively, and uses a wavelength of 1050 nm with a scan speed of 100,000 A-scans per second. This

© 2023 Yang S et al. *JAMA Network Open.*

instrument is characterized by high scanning speed and deep penetration, which is especially suitable for imaging of deep structures. Each OCT scan is performed through an internal fixator, and the fixation is monitored by the instrument's built-in fundus camera. Within 1.3 seconds, the retina was imaged using a 3D Macula Cube 7×7 mm scan mode centered on the fovea with a scan density of 512 A-scans × 512 B-scans.

The SS-OCT software automatically segments each layer of the retina and automatically measures the thickness of GCIPL. All OCT scans are performed by a trained technician who is unaware of the patient's diagnosis. Previous studies have demonstrated excellent reliability and reproducibility of both retinal and choroidal thickness measurements by SS-OCT. The results of the automated segmentation were evaluated by an image expert (WW), and the participants' data were masked during processing. If segmentation errors existed, manual adjustments were performed. Only high-quality scanned images were used for analysis, and images with (1) image quality scores <60, (2) defocus, (3) motion artifacts or blink artifacts, (4) out of center, (5) images with poor contrast due to refractive media opacity (local signal missing, blurred images, masking), and (6) unable-correctable segmentation errors were excluded.

Profiling details and quality control processes of LC/MS profiling

The sample stored at -80 °C refrigerator was thawed on ice and vortexed for 10 s. 50 µL of sample and 300 µL of extraction solution (ACN : Methanol = 1:4, V/V) containing internal standards were added into a 2 mL microcentrifuge tube. The sample was vortexed for 3 min and then centrifuged at 12000 rpm for 10 min (4 °C). 200 µL of the supernatant was collected and placed in -20 °C for 30 min, and then centrifuged at 12000 rpm for 3 min (4 °C). A 180 µL aliquots of supernatant were transferred for LC-MS analysis.

The sample extracts were analyzed using an LC-ESI-MS/MS system (UPLC, ExionLC

© 2023 Yang S et al. *JAMA Network Open.*

AD, https://sciex.com.cn/; MS, QTRAP® System, https://sciex.com/). The analytical conditions were as follows, UPLC: column, Waters ACQUITY UPLC HSS T3 C18 (1.8 µm, 2.1 mm*100 mm); column temperature, 40 °C; flow rate, 0.4 mL/min; injection volume, 2µL; solvent system, water (0.1% formic acid): acetonitrile (0.1% formic acid); gradient program, 95:5 V/V at 0 min, 10:90 V/V at 11.0 min, 10:90 V/V at 12.0 min, 95:5 V/V at 12.1 min, 95:5 V/V at 14.0 min.

LIT and triple quadrupole (QQQ) scans were acquired on a triple quadrupole-linear ion trap mass spectrometer (QTRAP), QTRAP® LC-MS/MS System, equipped with an ESI Turbo Ion-Spray interface, operating in positive and negative ion mode, and controlled by Analyst 1.6.3 software (Sciex). The ESI source operation parameters were as follows: source temperature 500 °C; ion spray voltage (IS) 5500 V (positive), -4500 V (negative); ion source gas I (GSI), gas II (GSII), curtain gas (CUR) were set at 55, 60, and 25.0 psi, respectively; the collision gas (CAD) was high. Instrument tuning and mass calibration were performed with 10 and 100 µmol/L polypropylene glycol solutions in QQQ and LIT modes, respectively. A specific set of MRM transitions were monitored for each period according to the metabolites eluted within this period.

Quality control samples (QC) were prepared by mixing sample extracts to analyze the reproducibility of the samples under the same processing method. During the instrument analysis process, one QC sample was inserted every ten analytical samples to monitor the reproducibility of the analysis process. Overlapping analysis of the total ion chromatogram (TIC) of different QC samples were used to evaluate the reproducibility of metabolite extraction and detection, i.e., technical reproducibility. The high stability of the instrument provides important guarantees for the reproducibility and reliability of the data.

To monitor the presence of any residual substances during the detection process, blank samples were included at various stages of the experiment. The appearance

© 2023 Yang S et al. *JAMA Network Open.*

of peaks in these samples could indicate cross-contamination between samples or the presence of unwanted substances.

In addition, to ensure the reliability and accuracy of the experimental results, internal standards with known concentrations were added to the quality control process. The response of the internal standards was used as an indicator of the stability of the detection process, with smaller variation in response indicating higher data quality. The CVs for all internal standards in the current profiling were all below 0.01.

Reference

1. Chen W, Gong L, Guo Z, et al. A Novel Integrated Method for Large-Scale Detection, Identification, and Quantification of Widely Targeted Metabolites: Application in the Study of Rice Metabolomics[J]. Molecular Plant, 2013, 6(6):1769-1780.
2. Fraga, C.G., et al., Signature-discovery approach for sample matching of a nerve-agent precursor using liquid chromatography-mass spectrometry, XCMS, and chemometrics. Anal Chem, 2010. 82(10): p. 4165-73.
3. Viant M R, Kurland I J, Jones M R, et al. How close are we to complete annotation of metabolomes? Current opinion in chemical biology, 2017, 36: 64-69.

Testing of the PH assumption and managing potential violations

First, the cumulative risk graph shows that all curves are roughly in line with proportional risk, except for other mortality. Then, the PH assumption was tested using the Schoenfeld residual method. Assuming risk varies over time, the function of the hazard ratio over time for any variable is expressed as follows: $\exp(\beta_i + \beta_{k+i} \times \phi(t))$. Thus, the expectation of the scaled Schoenfeld residuals for this variable at time point m is expressed as: $E(r^*_{Sch,m}) = \beta_{k+i} \times \phi(t)$. Therefore, to

test the PH hypothesis is equivalent to determining whether β_{k+i} is equal to zero. In the construction of the CPH models, interaction terms with time were introduced for variables that did not satisfy the PH assumption. If this variable was removed from subsequent stepwise regressions, the time interaction term for this variable was also removed along with it.

Details of the model development in the UKB cohort

We developed three models to discriminate each health outcome in the discovery set, namely a clinical indicators-based model, a GCIPLT metabolic state model, and a combined model. Feature selection was performed using LASSO, which achieves sparsity of model parameters by adding an L1 regularization term to the optimization objective function to reduce model complexity and the risk of overfitting. This approach is very effective when dealing with high-dimensional data, such as omics data. The selected variables were used to construct CPH models. After the Schoefeld residual method test described above, the necessary time interaction terms were added to the models. Stepwise regression was used to select models with the best performance, with Akaike information as the selection criterion and the direction of stepwise regression set to 'both'.

Details of the GCIPLT metabolic state models for each outcome are available in Supplementary eTable S2. The clinical indicators used for each outcome are as follows. The clinical indicators-based model for predicting T2D includes age, sex, Townsend deprivation index, smoking, drinking, ethnicity, BMI, WHR, blood pressure-lowering medication, and family history of diabetes. The clinical indicators-based model for predicting MI includes age, sex, ethnicity, smoking, drinking, BMI, hypertension, diabetes, total cholesterol, and LDL-c. The clinical indicators-based model for predicting HF includes age, sex, ethnicity, smoking, drinking, BMI, ACR, HbA1c, hemoglobin, cardiovascular diseases, and family history of CVD. The clinical indicators-based model for predicting stroke includes age, sex, ethnicity, smoking, drinking, BMI, hypertension, family history of stroke, total

© 2023 Yang S et al. *JAMA Network Open.*

cholesterol, and SBP. The clinical indicators-based model for predicting dementia includes age, sex, ethnicity, smoking, drinking, education, BMI, stroke, family history of dementia, and apoE4 allele. The clinical indicators-based model for predicting OSAHS includes age, sex, smoking, drinking, ethnicity, BMI, WHR, hypertension, and diabetes. The clinical indicators-based model for predicting mortality includes age, sex, Townsend deprivation index, smoking, drinking, BMI, physical activity, lipid-lowering medication, SBP, diabetes, and total cholesterol.

Methods for assessing predictive performance and clinical utility

Several metrics for assessing model performance were used in the current study. The C-statistic, also referred to as the concordance statistic, is a widely used statistical measure in medical research to evaluate the performance of predictive models. It assesses the ability of a diagnostic test or predictive model to distinguish between individuals with and without a specific outcome, such as a diseases or mortality. It ranges from 0.5 to 1.0, where a value of 0.5 indicates that the model has no discriminatory power and is no better than random guessing, while a value of 1.0 indicates perfect discrimination, meaning the model can correctly predict all cases. Typically, a C-statistic of 0.7 or higher is considered to indicate good performance.

The net reclassification index (NRI) is a statistical measure commonly used in medical research to assess the improvement in risk prediction models when new biomarkers or variables are added. The NRI evaluates the ability of a new model to correctly reclassify individuals into higher or lower risk categories compared to the original model. The NRI is calculated as the sum of two components: the proportion of individuals who are correctly reclassified into a higher risk category (NRI+) minus the proportion of individuals who are incorrectly reclassified into a lower risk category (NRI-), with a positive value indicating an improvement in risk prediction and a negative value indicating a worsening of risk prediction. Unlike other measures of model performance, such as the area under the receiver operating

© 2023 Yang S et al. *JAMA Network Open.*

characteristic curve (AUC), the NRI provides information on the direction and magnitude of reclassification, enabling direct comparison of the performance of two models, even when their AUCs are similar.

The calibration plot is a graphical tool that compares the predicted probabilities of a statistical model with the actual observed probabilities. It is commonly used in binary classification problems where the goal is to predict the probability of an event occurring or not occurring. The x-axis of the plot represents the predicted probabilities, while the y-axis represents the observed probabilities of the event occurring. The primary objective of a calibration plot is to assess the accuracy and reliability of a predictive model. Ideally, a well-calibrated model should have predicted probabilities that match the observed probabilities, resulting in a diagonal line with a slope of 1.0. Any deviation from this diagonal line indicates that the model is either overestimating or underestimating the probability of the event occurring. In practice, it is rare to achieve perfect calibration, and therefore the goal is to create a model that is as calibrated as possible. The calibration plot can be used to compare the calibration of different models by plotting their calibration curves on the same graph. The model with the closest fit to the diagonal line is considered as the most calibrated.

The decision curve analysis (DCA) is a statistical method that evaluates the clinical usefulness of predictive models and diagnostic tests. It was developed as an extension of the traditional ROC curve and net benefit analysis. DCA helps to determine whether a predictive model or diagnostic test has clinical value by assessing the net benefit of using the model or test compared to other available strategies for making decisions. The DCA method involves plotting the net benefit of a model or test against the threshold probability for making the decision. The threshold probability represents the probability of an event occurring at which the clinician would be willing to make a particular decision. The DCA plot displays the net benefit curves for the model or test being evaluated and for two other

© 2023 Yang S et al. *JAMA Network Open.*

reference strategies: a treat-all strategy and a treat-none strategy. The treat-all strategy assumes that all patients have the disease or outcome of interest and are treated, while the treat-none strategy assumes that no patients have the disease or outcome and do not receive treatment.

© 2023 Yang S et al. *JAMA Network Open.*

eAppendix

Extended discussion on T2D

GCIPLT metabolomic fingerprints provided an impressive value in the prediction of T2D. This study implicates decreased HDL cholesterol, and changes in its multiple components as factors that confer an increased risk of T2D, consistent with the complex shifts in lipid metabolism that occur in T2D. Precisely, the synthesis and release of triglyceride (TG)-rich very low-density lipoprotein (VLDL) in the liver drives the exchange of TG and cholesterol esters between VLDL and HDL, leading to a decrease in HDL cholesterol in T2D.[1] In addition, unstable TG-rich HDL particles are considered more susceptible to clearance,[2] explaining the negative association between HDL and apoA1 levels and the risk of developing T2D in this study. Since HDL particles are primarily responsible for reverse transport of cellular cholesterol, their reduction or inactivity can cause lipid accumulation in pancreatic β-cells, causing inflammation and impaired β-cell function.[3-6] This cholesterol transport activity and antioxidant capacity of HDL were also reported to be associated with its surface lipid components, which determines the protein's mobility[7-9]; hence, decreased ratios of unsaturated FAs to saturated FAs, phospholipids, and free cholesterol observed in the present study impair antioxidant capacity, which predispose an individual to T2D.

Extended discussion on dementia

While there was no significant improvement in the C-index, the GCIPLT metabolic fingerprints showed a significant increase in both the net reclassification power and clinical utility for dementia prediction. ApoA1, HDL particle concentration and multiple components within HDL were found to be independently associated with decreased risk of dementia. Debate about the prospective relationship between plasma HDL levels and dementia is ongoing,[10] and it was recently demonstrated that plasma apoA1-HDL crossed the blood–brain barrier through scavenger receptors, inferring it could participate in brain lipid metabolism.[11] Extracellular deposition of amyloid β (Aβ) is thought to be the initiating event for dementia,[12]

© 2023 Yang S et al. *JAMA Network Open.*

and *in vitro* studies show that apoA1 binds to Aβ to interfere with Aβ monomer assembly, preventing neurotoxicity.[13, 14] In addition, Aβ-bound HDL was reported to promote *in situ* degradation of Aβ by binding to scavenger receptors on glial cells,[15] explaining why plasma apoA1 and HDL were protective against dementia in this study. These consolidate the current literature and provide further insight into the pathophysiology of dementia.

Extended discussion on OSAHS

OSAHS is a common sleep disorder and airway disease characterized by sleep apnea, causing subsequent chronic hypoxia.[16] Elevated levels of free FAs (FFAs) are common in mice exposed to intermittent hypoxia (IH) and in OSAHS patients,[17-21] likely from IH-associated activation of the sympathetic system that triggers the release of FFAs from adipose tissue.[22] Similar to T2D, FFAs are sent to the liver to synthesize TG-rich VLDL, which ultimately decreases HDL cholesterol and clearance of unstable HDL particles,[2] as supported by our results. IH is also thought to impair the sensitivity of adipose tissue to insulin, which further leads to increased FFAs release and decreased HDL cholesterol and HDL particles.[23] In addition, patients with OSAHS have been reported to undergo lipid peroxidation more frequently,[24] hence higher levels of HDL particles with higher phospholipid and free cholesterol content that are associated with good antioxidant capacity were deemed protective against OSAHS in the present study.[8, 9]

Extended discussion on metrics used for model assessment

To ensure a further comprehensive evaluation of model performance and effectiveness in future real world-setting studies, it is essential to consider multiple indicators beyond the metrics used in the current study. Factors such as interpretability, robustness, and generalizability may also play a critical role in evaluating the model's quality. For instance, model interpretability refers to the ability to understand and explain the model's decision-making process, which can facilitate its adoption and implementation in real-world settings. Robustness, on the

© 2023 Yang S et al. *JAMA Network Open.*

other hand, refers to the model's ability to perform well even when the data is noisy or incomplete. Generalizability refers to the model's ability to perform well on data that it has not been trained on. By considering these factors, a more comprehensive and accurate assessment of the model's quality can be achieved.

References:

1. Kontush A, Chapman MJ. Why is HDL functionally deficient in type 2 diabetes? *Curr Diab Rep.* 2008;8(1):51-59.
2. Lewis GF, Rader DJ. New insights into the regulation of HDL metabolism and reverse cholesterol transport. *Circ Res.* 2005;96(12):1221-1232.
3. Kennedy MA, Barrera GC, Nakamura K, et al. ABCG1 has a critical role in mediating cholesterol efflux to HDL and preventing cellular lipid accumulation. *Cell Metab.* 2005;1(2):121-131.
4. Zhu X, Lee JY, Timmins JM, et al. Increased cellular free cholesterol in macrophage-specific Abca1 knock-out mice enhances pro-inflammatory response of macrophages. *J Biol Chem.* 2008;283(34):22930-22941.
5. Sun Y, Ishibashi M, Seimon T, et al. Free cholesterol accumulation in macrophage membranes activates Toll-like receptors and p38 mitogen-activated protein kinase and induces cathepsin K. *Circ Res.* 2009;104(4):455-465.
6. Drew BG, Rye KA, Duffy SJ, Barter P, Kingwell BA. The emerging role of HDL in glucose metabolism. *Nat Rev Endocrinol.* 2012;8(4):237-245.
7. Yancey PG, de la Llera-Moya M, Swarnakar S, et al. High density lipoprotein phospholipid composition is a major determinant of the bi-directional flux and net movement of cellular free cholesterol mediated by scavenger receptor BI. *J Biol Chem.* 2000;275(47):36596-36604.
8. Zerrad-Saadi A, Therond P, Chantepie S, et al. HDL3-mediated inactivation of LDL-associated phospholipid hydroperoxides is determined by the redox status of apolipoprotein A-I and HDL particle surface lipid rigidity: relevance to inflammation and atherogenesis. *Arterioscler Thromb Vasc Biol.* 2009;29(12):2169-2175.

© 2023 Yang S et al. *JAMA Network Open.*

9. Vila A, Korytowski W, Girotti AW. Spontaneous transfer of phospholipid and cholesterol hydroperoxides between cell membranes and low-density lipoprotein: assessment of reaction kinetics and prooxidant effects. *Biochemistry-Us.* 2002;41(46):13705-13716.
10. Koch M, Jensen MK. HDL-cholesterol and apolipoproteins in relation to dementia. *Curr Opin Lipidol.* 2016;27(1):76-87.
11. Jin Y, Chifodya K, Han G, et al. High-density lipoprotein in Alzheimer's disease: From potential biomarkers to therapeutics. *J Control Release.* 2021;338:56-70.
12. Lane CA, Hardy J, Schott JM. Alzheimer's disease. *Eur J Neurol.* 2018;25(1):59-70.
13. Merino-Zamorano C, Fernandez-de RS, Montanola A, et al. Modulation of Amyloid-beta1-40 Transport by ApoA1 and ApoJ Across an in vitro Model of the Blood-Brain Barrier. *J Alzheimers Dis.* 2016;53(2):677-691.
14. Paula-Lima AC, Tricerri MA, Brito-Moreira J, et al. Human apolipoprotein A-I binds amyloid-beta and prevents Abeta-induced neurotoxicity. *Int J Biochem Cell Biol.* 2009;41(6):1361-1370.
15. Sagare AP, Bell RD, Zlokovic BV. Neurovascular dysfunction and faulty amyloid beta-peptide clearance in Alzheimer disease. *Cold Spring Harb Perspect Med.* 2012;2(10).
16. Gottlieb DJ, Punjabi NM. Diagnosis and Management of Obstructive Sleep Apnea: A Review. *JAMA.* 2020;323(14):1389-1400.
17. Jun J, Reinke C, Bedja D, et al. Effect of intermittent hypoxia on atherosclerosis in apolipoprotein E-deficient mice. *Atherosclerosis.* 2010;209(2):381-386.
18. Weiszenstein M, Shimoda LA, Koc M, Seda O, Polak J. Inhibition of Lipolysis Ameliorates Diabetic Phenotype in a Mouse Model of Obstructive Sleep Apnea. *Am J Respir Cell Mol Biol.* 2016;55(2):299-307.
19. Barcelo A, Pierola J, de la Pena M, et al. Free fatty acids and the metabolic syndrome in patients with obstructive sleep apnoea. *Eur Respir J.* 2011;37(6):1418-1423.
20. Jun JC, Drager LF, Najjar SS, et al. Effects of sleep apnea on nocturnal free fatty acids in subjects with heart failure. *Sleep.* 2011;34(9):1207-1213.

© 2023 Yang S et al. *JAMA Network Open.*

21. Chopra S, Rathore A, Younas H, et al. Obstructive Sleep Apnea Dynamically Increases Nocturnal Plasma Free Fatty Acids, Glucose, and Cortisol During Sleep. *J Clin Endocrinol Metab.* 2017;102(9):3172-3181.
22. Lafontan M, Langin D. Lipolysis and lipid mobilization in human adipose tissue. *Prog Lipid Res.* 2009;48(5):275-297.
23. Wood IS, de Heredia FP, Wang B, Trayhurn P. Cellular hypoxia and adipose tissue dysfunction in obesity. *Proc Nutr Soc.* 2009;68(4):370-377.
24. Barcelo A, Miralles C, Barbe F, Vila M, Pons S, Agusti AG. Abnormal lipid peroxidation in patients with sleep apnoea. *Eur Respir J.* 2000;16(4):644-647.

© 2023 Yang S et al. *JAMA Network Open.*
