# Supplementary material for: Analysis of Plasma Metabolic Profile on Ganglion Cell–Inner Plexiform Layer Thickness With Mortality and Common Diseases
Source: JAMA Netw Open. 2023 May 16;6(5):e2313220. doi: 10.1001/jamanetworkopen.2023.13220 (PMC10189567; doi:10.1001/jamanetworkopen.2023.13220)
Supplement: Supplement 2. — Data Sharing Statement [file jamanetwopen-e2313220-s002.pdf]

Data Sharing Statement

Yang. Analysis of Plasma Metabolic Profile on Ganglion Cell–Inner Plexiform Layer Thickness With Mortality and Common Diseases. *JAMA Netw Open*. Published May 16, 2023.
doi:10.1001/jamanetworkopen.2023.13220

Data
Data available: Yes
Data types: Deidentified participant data
How to access data: http://www.ukbiobank.ac.uk
When available: With publication

Supporting Documents
Document types: None

Additional Information
Who can access the data: Researchers whose proposed use of the data has been approved
Types of analyses: For research purposes.
Mechanisms of data availability: After approval of a proposal.
